# Natural Stibnite for Lithium-/Sodium-Ion Batteries: Carbon Dots Evoked High Initial Coulombic Efficiency

**DOI:** 10.1007/s40820-022-00873-x

**Published:** 2022-06-17

**Authors:** Yinger Xiang, Laiqiang Xu, Li Yang, Yu Ye, Zhaofei Ge, Jiae Wu, Wentao Deng, Guoqiang Zou, Hongshuai Hou, Xiaobo Ji

**Affiliations:** 1 College of Chemistry and Chemical Engineering, Central South University, Changsha, 410083 People’s Republic of China; 2 College of Science, Hunan University of Technology and Business, Changsha, 410205 People’s Republic of China; 3 School of Material Science and Engineering, Zhengzhou University, Zhengzhou, 450001 People’s Republic of China

**Keywords:** Carbon dots, Sb_2_S_3_, Initial Coulombic efficiency, Interfacial bond, Anode

## Abstract

**Highlights:**

- The chemical process of local oxidation–partial reduction–deep coupling for stibnite reduction of carbon dots (CDs) is revealed by in-situ high-temperature X-ray diffraction.
- Sb_2_S_3_@xCDs anode delivers high initial coulombic efficiency in lithium ion batteries (85.2%) and sodium ion batteries (82.9%), respectively.
- C–S bond influenced by oxygen-rich carbon matrix can restrain the conversion of sulfur to sulfite, well confirmed by X-ray photoelectron spectroscopy characterization of solid electrolyte interphase layers helped with density functional theory calculations.
- CDs-induced Sb–O–C bond is proved to effectively regulate the interfacial electronic structure.

**Abstract:**

The application of Sb_2_S_3_ with marvelous theoretical capacity for alkali metal-ion batteries is seriously limited by its poor electrical conductivity and low initial coulombic efficiency (ICE). In this work, natural stibnite modified by carbon dots (Sb_2_S_3_@xCDs) is elaborately designed with high ICE. Greatly, chemical processes of local oxidation–partial reduction–deep coupling for stibnite reduction of CDs are clearly demonstrated, confirmed with in situ high-temperature X-ray diffraction. More impressively, the ICE for lithium-ion batteries (LIBs) is enhanced to 85%, through the effect of oxygen-rich carbon matrix on C–S bonds which inhibit the conversion of sulfur to sulfite, well supported by X-ray photoelectron spectroscopy characterization of solid electrolyte interphase layers helped with density functional theory calculations. Not than less, it is found that Sb–O–C bonds existed in the interface effectively promote the electronic conductivity and expedite ion transmission by reducing the bandgap and restraining the slip of the dislocation. As a result, the optimal sample delivers a tremendous reversible capacity of 660 mAh g^−1^ in LIBs at a high current rate of 5 A g^−1^. This work provides a new methodology for enhancing the electrochemical energy storage performance of metal sulfides, especially for improving the ICE.
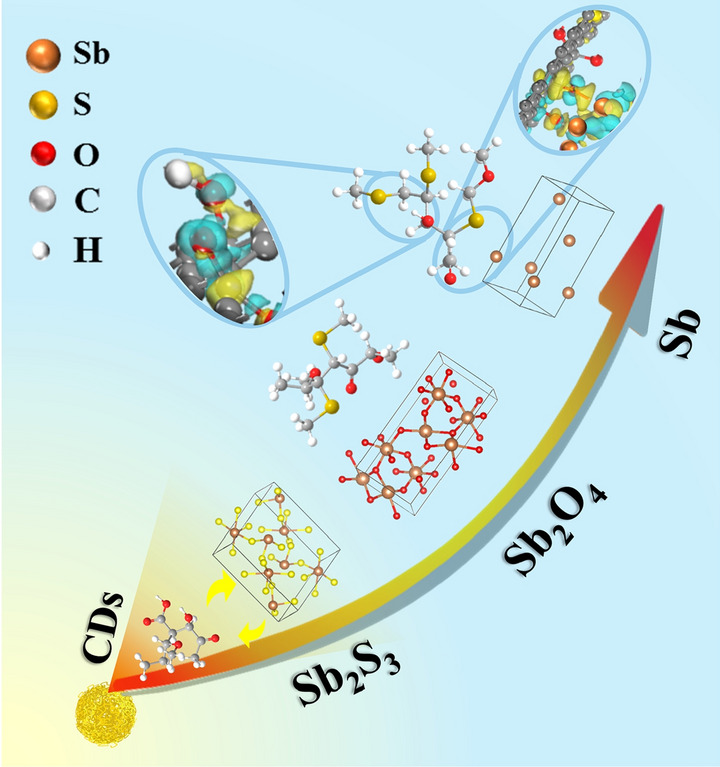

**Supplementary Information:**

The online version contains supplementary material available at 10.1007/s40820-022-00873-x.

## Introduction

Antimony sulfide (Sb_2_S_3_) has attracted tremendous attentions for advanced energy storage due to its high theoretical storage capacity (~ 947 mAh g^−1^), suitable electrode potential, and low cost [1]. Nevertheless, the practical application of Sb_2_S_3_ suffers from poor conductivity, weak structural stability, lower initial Coulombic efficiency (ICE), and “shuttling effect” of polysulfide [2, 3]. In order to tackle these key problems, multifarious ways have been proposed, such as combining with highly conductive carbonaceous materials [4, 5], designing greatly stable structures [6, 7], building interfacial bonds [8], and introducing doping heteroatoms [9]. Constructing Sb_2_S_3_/carbon composite is the most common and effective strategy [10–12]. For instance, Sb_2_S_3_/sulfur-doped graphene sheets composite prepared by Liu et al. showed a high-capacity retention of 83% for 900 cycles at 2 A g^−1^ [13]. And Sb_2_S_3_ with double carbon (M-Sb_2_S_3_@DC) designed by Sun et al. meaningfully verified that the existing Sb–C bond strengthened ions transfer and the controlling of Sb and S, resulting in an excellent rate capacity (674 mAh g^−1^ at 5 A g^−1^) [14]. It should be noted that the ICE is the key index of the performance of the full battery, and only the electrode materials with ICE can be practical. However, although the cycling stability and rate capability are improved, ICE is too low (< 70%) to be practical. Unfortunately, until now, the poor ICE of metal sulfides is still a tough nut to crack, and in-depth systematic research is quite insufficient. As is well known, the low ICE is mainly caused by the formation of solid electrolyte interphase (SEI) and irreversible side reaction, which is closely related to the interface characteristics between electrode materials and electrolyte [15]. Therefore, in the same electrolyte, the surface structure and composition of electrode materials directly determine the ICE [16]. Designing rational electrode materials, which can induce the formation of stable SEI layer and minimize side reactions, is the ideal strategy to obtain high ICE.

Owing to ultrasmall size (< 10 nm) and abundant functional groups, carbon dots (CDs) have quite unique physicochemical properties [17]. In our previous study, it was proved that CDs can be converted into functional carbon materials after heat treatment [18], which exhibited superior electrochemical performance. Therefore, we propose an approach to construct high-performance Sb_2_S_3_/carbon composites by calcining the low-cost natural stibnite and CDs. Clearly, it was found that the introduction of CDs can enhance the cyclic stability and rate performance of Sb_2_S_3_ and significantly improve the ICE. In the process of heat treatment, Sb_2_S_3_ can be utilized as sulfur sources to introduce into CDs-derived carbon matrix and form C-S bonds. In combination with experimental results and theoretical calculations, Sb_2_S_3_@xCDs could simultaneously settle matters in enhancing the intrinsic sluggish kinetics and ICE. Firstly, huge amounts of laminar carbon sheets in the outer of Sb_2_S_3_ ensure efficient channels for electron/ion transport and buffer for volume expansion; additionally, the chemical Sb–O–C between the reduced antimony and CDs-derived carbon matrix at the heterointerface contributes to the electron transportation and ions diffusion; and various oxygen-containing functional groups in the surface of CDs-derived carbon matrix converge “charge sea” which can restrain the formation of sulfites; finally, Sb_2_S_3_@xCDs at different degrees of reduction can be obtained through adjusting and controlling the ratio of Sb_2_S_3_ and CDs. As a result, when applied as an anode, Sb_2_S_3_@xCDs shows a high ICE (85% in lithium-ion batteries (LIBs), 83% in sodium-ion batteries (SIBs)) and an excellent reversible capacity of 660 mAh g^−1^ in LIBs at a high current rate of 5 A g^−1^. Moreover, the Li-storage capacity can be remained about 648.1 mAh g^−1^ after 100 cycles at the current density of 0.1 A g^−1^.

## Experimental Section

### Materials and Methods

#### ***Synthesis of Sb***_***2***_***S***_***3***_*** Powder***

Natural stibnite was obtained from Guangzhou Huadu Ye’s Stone Specimen Firm. Firstly, natural stibnite was roughly smashed for 6 h with a three-head grinding machine. Then the stibnite was ball milled at 400 r min^−1^ for 4 h to acquire stibnite powder, denoted as stibnite Sb_2_S_3_ (contrast sample).

#### Synthesis of CDs

The synthesis of CDs was based on our previous reports [19]. NaOH (12 g) was slowly added to acetaldehyde (40%, 40 mL), and kept magnetically stirring for 2 h. Afterward, the obtained-solid was sonicated with 1 M HCl solution and deionized water until it became neutral and flocculent. Finally, products were collected in the blast drying oven at a lower temperature.

#### ***Synthesis of Sb***_***2***_***S***_***3***_***@xCDs***

Sb_2_S_3_ powder and CDs were mixed adequately and evenly by mass ratio of 1:0.1, 1:0.3, and 1:0.5, and then kept at 700 °C (10 °C min^−1^) for 5 h under high purity argon atmosphere. The final products recorded as Sb_2_S_3_@xCDs (x means the content of CDs) were the requested electrode materials.

### Materials Characterization

Structures of Sb_2_S_3_ and Sb_2_S_3_@xCDs were characterized by X-ray diffraction (XRD) with a Cu-Kα radiation (λ = 1.54 Å) operating at 40 kV and 250 mA as well as the phase transition and formation of Sb_2_S_3_@xCDs was carried by the HT-XRD (Rigaku, TTR3, 40 kV, Cu-Kα radiation). The microstructures of samples were explored by scanning electron microscopy (SEM, Magellan 400) and transmission electron microscopy (TEM, JEOL JEM 2100F). Raman spectra were recorded on Raman spectroscopy (Renishaw) with the 532 nm laser excitation. Thermo Scientific EscaLab 250Xi and Fourier-transform infrared (FT-IR) spectra were used to reveal the surface chemical bonds of the samples. Specific surface area, pore size distributions, and pore volume of samples were determined by the N_2_ adsorption–desorption isotherms through a Micromeritics ASAP 2020 instrument and calculated by the Brunauer–Emmett–Teller (BET) equation, respectively. The TGA was measured by a Netzsch-STA449F5 in air atmospheres at a heating rate of 10 °C min^−1^.

### Electrochemical Measurements

In order to obtain electrode for electrochemical test, slurry was composed of 70 wt% active materials, 15 wt% carboxymethyl cellulose (CMC), and 15 wt% acetylene black (Super P) in deionized water. After the slurry was well mixed, it was coated on copper foil and dried in vacuum at 80 °C for 10 h. For the preparation of Li^+^/Na^+^ half cells, the working electrodes, the counter (lithium metal/sodium metal), electrolyte (1.0 M LiPF_6_ in EC:DMC:DEC = 1:1:1 Vol% with 5.0% FEC/1.0 M NaPF_6_ in EC:DMC:DEC = 1:1:1 Vol% with 5.0% FEC), and separator (polypropylene membrane) were assembled to coin cells (CR2016) in the argon-filled glovebox (both H_2_O and O_2_ < 0.5 ppm). Galvanostatic charge/discharge tests were measured by LAND CT 2001A. Cyclic voltammetry (CV) curves were implemented by MULTI AUTO LAB M204, with the voltage range between 0.01 and 3.00 V (vs Li/Li^+^/ Na/Na^+^). Electrochemical impedance spectra (EIS) were collected on MULTI AUTOLAB M204 (MAC90086).

### Theoretical Computation

The Vienna Ab Initio Package (VASP) [20] was utilized to achieve the density functional theory (DFT) calculations [21] which was combined with the Perdew, Burke, and Ernzerhof (PBE) in the generalized gradient approximation (GGA) [22]. The ionic cores were described by the projected augmented wave (PAW) potentials [23] and valence electrons were considered by using a plane wave basis set with a kinetic energy cutoff of 450 eV [24]. Using the Gaussian smearing method can occupy some orbitals of the Kohn–Sham with a width of 0.05 eV. When the energy change was below 10 ^− 4^ eV, the electronic energy was regarded self-consistent. And when the change of force was smaller than 0.03 eV Å^−1^, geometry optimization was equivalent to convergent. The dispersion interactions were performed by Grimme’s DFT-D3 methodology [25]. After taking advantage of a 4 × 4 × 2 Monkhorst–Pack k-point grid for Brillouin zone sampling, there was a reasonable optimization for the equilibrium lattice constants of Sb_2_S_3_ and Sb unit cell. The Climbing Image-Nudged Elastic Band methods had been employed to calculate the migration barriers of Li^+^ ions in the Sb_2_S_3_ and Sb_2_S_3_@xCDs structures.

## Results and Discussion

### Structural Analysis

**Fig. 1:**
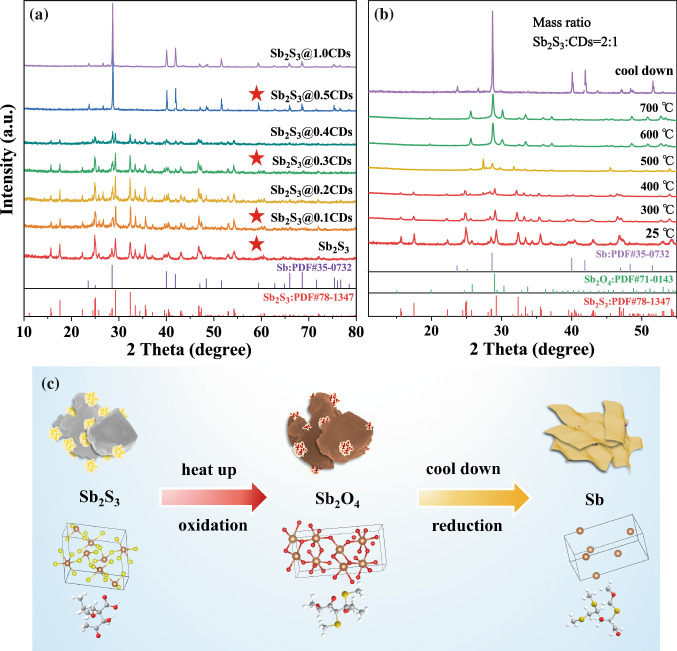
Phase and structure characterization. **a** XRD patterns of Sb_2_S_3_@xCDs and Sb_2_S_3_. **b** HT-XRD for Sb_2_S_3_@0.5CDs from 25 to 700 °C under nitrogen atmosphere with a heating rate of 10 °C min^−1^. **c** Schematic for the formation process of Sb_2_S_3_@xCDs

Sb_2_S_3_@xCDs composites with different Sb/C ratios are elaborately prepared by adjusting the ratio of stibnite and CDs in the heat treatment process. XRD is conducted on natural stibnite and Sb_2_S_3_@xCDs hybrids to figure out their crystal structures in Fig. 1a. The diffraction peaks of stibnite are indexed to standard orthorhombic phase Sb_2_S_3_ (PDF No. 78-1347). As the amounts of CDs increase, Sb_2_S_3_ is gradually reduced to Sb and the peak corresponding to the (110) lattice planes of Sb (PDF No. 35-0732) at around 41.9° gets more and more sharp. When the mass ratio of Sb_2_S_3_ and CDs runs up to 2, Sb_2_S_3_ is converted to metallic Sb completely. High-temperature XRD (HT-XRD) is utilized to explore the *in situ* evolution of crystallographic phases during annealing. Following the set program with a constant heating rate of 10 °C min^−1^ from 25 °C (room temperature) to 700 °C and then cooling down naturally, a series of XRD patterns is obtained. The initial XRD pattern of the mixture (the mass ratio of Sb_2_S_3_ and CDs is 2) shows four different diffraction peaks totally in Figs. 1b and S1. Below 400 °C, no other phase appears except antimony sulfide. When the temperature is beyond 500 °C, XRD patterns present mixture phase of Sb_2_S_3_ and Sb_2_O_4_ (PDF No. 71-0143), proving that a part of Sb_2_S_3_ is oxidized by oxygen-containing functional groups of CDs, and then the peak of Sb_2_S_3_ is gradually disappeared along with heating, Sb_2_S_3_ is converted to Sb_2_O_4_ completely. Eventually, Sb_2_O_4_ is reduced to Sb by the carbothermic reduction of CDs-derived carbon. Furthermore, a newly prepared potassium permanganate solution (KMnO_4_) is utilized to detect the gas formed in the heat treatment process (Fig. S2). The solution faded and precipitation is generated, which are verified to be S and MnO_2_ through XRD test (Fig. S3). Therefore, it can be judged that H_2_S gas is generated in the annealing reaction process and the following reaction occurred between H_2_S and KMnO_4_:

$${\text{2KMnO}}_{{4}} + {\text{ 3H}}_{{2}} {\text{S }} = {\text{ 2MnO}}_{{2}} \downarrow + {\text{ 2KOH }} + {\text{3S}} \downarrow + {\text{ 2H}}_{{2}} {\text{O}}{.}$$

In a word, the results above manifest that the reduction of Sb_2_S_3_ to metallic Sb experience processes of local oxidation, partial reduction, and deep coupling which looks like pyrometallurgy. And the schematic diagram displayed in Fig. 1c reveals the concrete procedures of Sb_2_S_3_@xCDs composites.

**Fig. 2:**
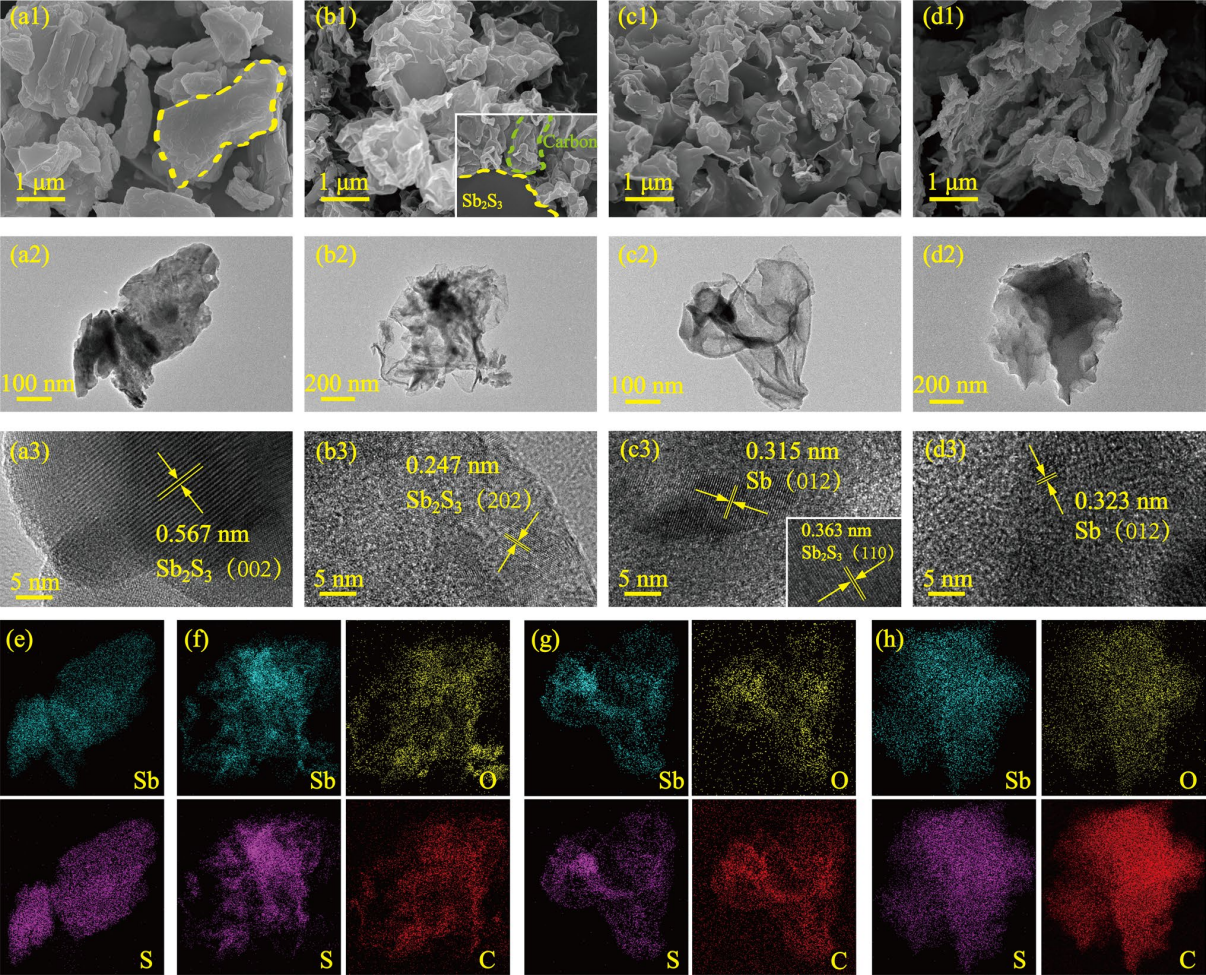
SEM, TEM, and HRTEM images of (**a**) stibnite Sb_2_S_3_, **b** Sb_2_S_3_@0.1CDs, **c** Sb_2_S_3_@0.3CDs and (**d**) Sb_2_S_3_@0.5CDs. The corresponding EDX element mapping images of (**e**) Sb_2_S_3_, **f** Sb_2_S_3_@0.1CDs, **g** Sb_2_S_3_@0.3CDs, and (**h**) Sb_2_S_3_@0.5CDs

**Fig. 3:**
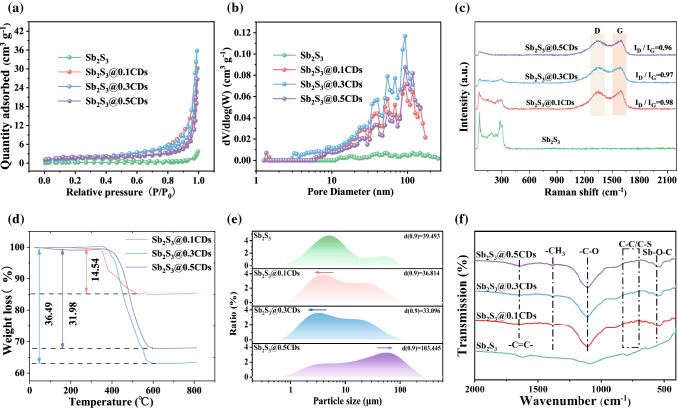
**a** Nitrogen adsorption–desorption isothermal curves. **b** The pore size distribution curves. **c** Raman spectra. **d** TGA curves. **e** Size distributions. **f** FT-IR spectra from 400 to 2000 cm^−1^

According to the result of XRD, Sb_2_S_3_, Sb_2_S_3_@0.1CDs, Sb_2_S_3_@0.3CDs, and Sb_2_S_3_@0.5CDs with representativeness are selected to explore more elaborate structural features among all samples. In Fig. 2, the detailed morphology and internal structure are investigated by scanning electron microscopy (SEM) and transmission electron microscopy (TEM). It is observed that thin and small carbon nanosheets generated by self-assembly of CDs are coated on stibnite in Fig. 2(b1). However, as the content of CDs is increased, nanosheets of Sb_2_S_3_@0.3CDs are adequately cross-linking together like tiles in Fig. 2(c1). When CDs continue to increase, the slight agglomeration of nanosheets can be observed which is corresponding to the result of particle sizes in Fig. 3e. The irregular and thick sheets around Sb_2_S_3_ play great roles in extending the polysulfide shuttle path and enhancing the electrical conductivity of composites [26]. In Fig. 2(a2–d2), Sb_2_S_3_ is gradually reduced to Sb with more CDs. Obviously, there is a core of Sb_2_S_3_ in the center of Sb_2_S_3_@0.1CDs and Sb_2_S_3_@0.3CDs surrounded by a thin layer of carbon which can stabilize structures availably and further buffer the volume expansion [27]. But for Sb_2_S_3_@0.5CDs, all Sb_2_S_3_ particles are drastically changed into Sb (Fig. 2(d2)). In the high-resolution TEM (HRTEM) image of stibnite, the distance of lattice spacing is calculated about 0.567 nm, indexed to the (002) planes of Sb_2_S_3_ (PDF No. 78-1347). Then, in Fig. 2(b3), it is easy to find the lattice fringe of 0.247 nm, which is assigned to the (202) facet of Sb_2_S_3_, but no lattice information of Sb is observed, which may be ascribed to the quite low content of Sb in Sb_2_S_3_@0.1CDs. As shown in Fig. 2(c3), the lattice fringes of 0.315 nm and 0.363 nm correspond to the (012) facet of Sb and the (110) plane of Sb_2_S_3_ (PDF No. 35-0732), respectively, demonstrating that Sb_2_S_3_ and Sb coexist in the Sb_2_S_3_@0.3CDs composites. For Sb_2_S_3_@0.5CDs, as shown in Fig. 2(d3), Sb embedded in the carbon layer displays an obvious lattice fringe (0.323 nm), corresponding to the (012) plane. Besides, Fig. 2e–h displays the elemental mapping images of all elements in as-obtained samples, it is worth noting that Sb, S, O, and C are distributed uniformly in Sb_2_S_3_@0.1CDs, Sb_2_S_3_@0.3CDs, and Sb_2_S_3_@0.5CDs, oxygen element came from the oxygen-containing functional groups of CDs, and carbon element is from the CDs.

Pore structure and specific surface area have a nonnegligible influence on the electrochemical properties. As shown in Fig. 3a, N2 adsorption isotherm plots of four samples all show type-IV curves combined with an H3-type hysteresis loop [28]. The specific surface areas of Sb_2_S_3_@0.1CDs, Sb_2_S_3_@0.3CDs, and Sb_2_S_3_@0.5CDs are 5.88, 6.09, and 5.54 m^2^ g^−1^, which are much larger than that of stibnite Sb_2_S_3_ (0.88 m^2^ g^−1^). Focusing on Fig. 3b, Sb_2_S_3_@0.1CDs, Sb_2_S_3_@0.3CDs, and Sb_2_S_3_@0.5CDs deliver dominant pore distribution of about 100 nm, which are beneficial to promote the wettability of the electrolyte efficiently, boost ion diffusion availably and make rate performance better [29]. As depicted in Fig. 3c, Raman spectra measurement is conducted to analyze the local structure and carbon characteristic [19]. The peaks of Sb_2_S_3_ centered around 188 and 288 cm^−1^ are related to the vibration of S–Sb–S and the S–Sb stretching. Specially, the peak located in 299 cm^−1^ is closely associated with the symmetric vibration of the C_3v_ symmetric pyramidal Sb_2_S_3_ unit. Additionally, the peaks of 109 and 145 cm^−1^ are related to the *E*_g_ and *A*_1g_ bands of Sb [2], confirming the formation of Sb, agreeing well with the results of XRD. In the Raman spectrums of Sb_2_S_3_@0.1CDs, Sb_2_S_3_@0.3CDs, and Sb_2_S_3_@0.5CDs, there are two broad peaks of 1350 and 1590 cm^−1^ [30], representing the degree of defects (D-band) and graphitic structure (G-band) of carbon materials. The intensity ratios of D-band and G-band (*I*_D_/*I*_G_) become much less, indicating the weakened degree of graphitization with the increased CDs [31].

**Fig. 4:**
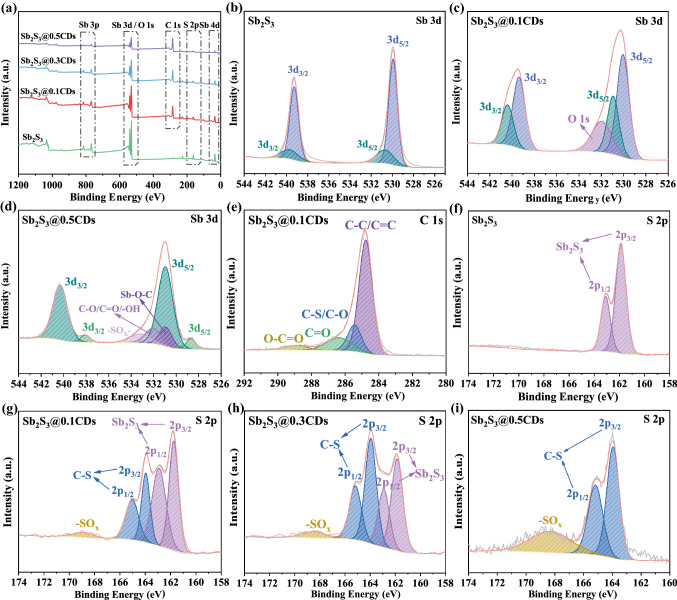
**a** Full XPS survey spectra. High-resolution XPS spectra of Sb 3*d* and S 2*p* of (**b, f**) stibnite Sb_2_S_3_, **c, e, g** Sb 3*d*, C 1*s*, and S 2*p* spectra of Sb_2_S_3_@0.1CDs, **h** S 2*p* spectra of Sb_2_S_3_@0.3CDs, and **d, i** Sb 3*d* and S 2*p* spectra of Sb_2_S_3_@0.5CDs

Thermogravimetric analysis (TGA) and X-ray fluorescence (XRF) test are performed to quantificationally calculate the content of Sb element. It is an essential method for natural mineral to ascertain specific components. And the antimony content of stibnite is 76.90% in Fig. S4. In TGA test (Fig. 3d), when heating as-obtained complexes in air atmosphere, carbon materials can become CO_2_ gas and Sb_2_S_3_/Sb will be changed into Sb_2_O_4_ (Fig. S5) drastically. By a series of calculations, the mass content of antimony in the Sb_2_S_3_@0.1CDs, Sb_2_S_3_@0.3CDs, and Sb_2_S_3_@0.5CDs are 68.19%, 51.13%, and 26.93%, respectively (the concrete processes of calculation are shown in the Supporting Information). As a vital indicator, particle size is relative with the distribution field of reaction active sites and the infiltration of electrolyte. Stibnite Sb_2_S_3_ can be reduced to particles of different sizes in the presence of CDs. But the excess CDs would lead to the agglomeration of carbon and Sb_2_S_3_/Sb, so the particle size distribution transfers to a high value. Figure S6 shows the electronic conductivity of four samples through the four-probe method. As a result, the conductivity of Sb_2_S_3_@xCDs is much higher than that of stibnite Sb_2_S_3_ due to the introduction of carbon and metallic Sb. To seek for the existed functional groups of composites, FT-IR is shown in Fig. 3f. The main bands of carbon are C=C (1600–1700 cm^−1^), –CH_3_ (1350–1420 cm^−1^), C–O (950–1260 cm^−1^), and C–C/C–S (690–840 cm^−1^). It is worth noting that the band at 600–550 cm^−1^ represents the vibration of Sb–O–C bonds, which proves the formation of electric/chemical coupling in Sb_2_S_3_@xCDs heterointerface. Thanks to rich functional groups, Sb_2_S_3_@xCDs composites provide abundant active sites for the storage and transmission of ions [32]. Except for FT-IR, X-ray photoelectron spectroscopy (XPS) is also performed to explore the chemical environment of related elements and their coupling effects in Figs. 4 and S7. Full survey scan spectrum (Fig. 4a) reveals the peaks of Sb, O, C, and S clearly. As for Sb 3*d* spectrum, peaks at 529.9 and 530.7 eV are related to Sb 3*d*_5/2_ and peaks at 539.3 and 539.7 eV are assigned to Sb 3*d*_3/2_ [33], indicating the presence of Sb^3+^ in stibnite (Fig. 4b). In Fig. 4c, peaks situated at 530.1 and 530.9 eV are ascribed to Sb 3*d*_5/2_ and peaks centered at 539.4 and 540.4 eV are regarded as Sb 3*d*_3/2_ [34]. The peak of O 1* s* introduced by CDs arises in the neighboring position of Sb 3*d*_5/2_ [35]. And the specific formation of O 1*s* is put in Fig. 4d, including Sb–O–C, C=O, C–O, –OH, and –SO_x_ bonds. The unusual peak at 530.9 eV further confirms the formation of Sb–O–C bonds in Sb/C interface [8]. As a profitable construction of interfacial chemical bond, Sb–O–C can be served as an amphibious bridge to enhance the fast transport of electron/ion as well as structure stability. Besides, as in Fig. 4d, the peaks located at 528.7 and 538.1 eV are attributed to the Sb^0^ [36], which reveals the existence of metallic Sb. Additionally, the peaks of C 1*s* (Fig. 4e) are deconvoluted into four main peaks of 284.8, 285.6, 286.6, and 288.6 eV, corresponding to C–C/C=C, C–S/C–O, C=O, and O–C=O [37], respectively, which may facilitate the formation of reliable heterointerface and unique “charge sea.” Associated with the HT-XRD results, the whereabouts of sulfur are clearly delineated in Fig. 4f–i. Under the action of CDs, sulfur is escaped from stibnite slowly and converted into H_2_S gas or hides in the carbon materials with C–S bond selectively. There is no Sb–S bond in the S spectrum anymore while CDs are excessive, proving that Sb_2_S_3_ is completely reduced, coinciding well with the results of XRD and TEM.

The subtlety of this study lies in the use of oxygen-containing carbon dots. In fact, in metallurgical research, metal sulfide cannot be reduced to metal by carbon. Generally, sulfide ore is first sintered in air to form oxide, and then further reduced to metal by carbothermic reduction. Here, the oxidation and reduction processes are completed in one sintering process, largely simplifying the preparation process of materials. The coexistence of Sb_2_S_3_, Sb, and carbon with interface chemical bond ensures the excellent properties of the composite. In particular, high specific capacity is guaranteed by Sb_2_S_3,_ the electrical conductivity involves a positive combination of Sb and carbon, and the structure stability of the composite is further enhanced by carbon matrix.

### Electrochemical Performance

**Fig. 5:**
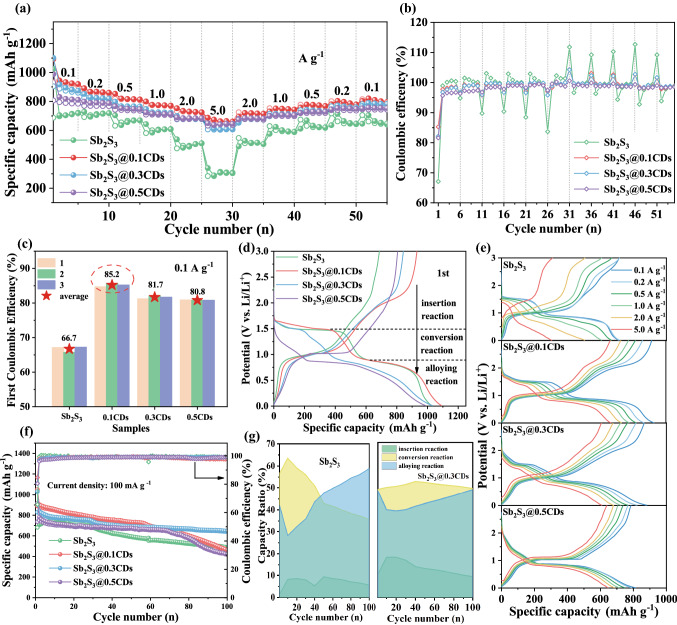
Lithium storage performance. **a** Rate capability and **b** Coulombic efficiency of the four samples at various current densities from 0.1 to 5 A g^−1^. **c** The initial Coulombic efficiency of the four electrodes at a density of 0.1 A g^−1^. **d** The first discharge/charge curves of the four samples. **e** Discharge/charge profiles of four electrodes at the corresponding rates. **f** Cycling performance of the four electrodes at a density of 0.1 A g^−1^. **g** The real-time capacity ratio of three diverse reactions when Sb_2_S_3_ and Sb_2_S_3_@0.3CDs anodes are discharged/charged at a current of 0.1 A g^−1^

The lithium storage performances of obtained samples were evaluated by assembling coin-type 2016 cell. The rate performance was further investigated at various current densities within the potential window of 0.01–3.0 V. As depicted in Fig. 5a, Sb_2_S_3_@xCDs electrodes exhibit obviously improved rate performance. When cycled at 0.1, 0.2, 0.5, 1, and 2 A g^−1^, the capacities of Sb_2_S_3_@0.1CDs are 920, 854, 808, 767, and 721 mAh g^−1^, respectively. At a high current density of 5 A g^−1^, the Sb_2_S_3_@0.1CDs delivers a percussive reversible capacity of 660 mAh g^−1^ specially. Comparing with Sb_2_S_3_@0.1CDs, the average charge capacities of stibnite Sb_2_S_3_ anode are only 710, 715, 667, 607, 505, and 304 mAh g^−1^ at the corresponding current densities, which are much lower than those of Sb_2_S_3_@xCDs anodes. Whenever there is a sudden change in the current density, Sb_2_S_3_@xCDs hybrids have high adaptability as well as a dramatic improvement in overcharging, which can be exactly reflected in the CE in Fig. 5b. In conclusion, the introduction of CDs can not only enhance the conductivity to speed up electron transfer, but also increase the ion transmission channels for active large current changes. In particular, in Fig. 5c, Sb_2_S_3_@xCDs anodes present a higher ICE than that of stibnite Sb_2_S_3_, which is a miracle in most antimony sulfide and even metal sulfides. Several parallel batteries tests are taken to eliminate the accidental errors for the four samples. The average ICE of Sb_2_S_3_@xCDs are 85.2% (Sb_2_S_3_@0.1CDs), 81.7% (Sb_2_S_3_@0.3CDs), and 80.8% (Sb_2_S_3_@0.5CDs), which are much higher than that of stibnite Sb_2_S_3_ (66.7%). In particular, the effective ways of enlarging the ICE in the first cycle are to avoid invalid electrolyte losses and to improve the reversibility of the charging–discharging reaction. Therefore, this is probably due to the unique “core-layer” structure, the carbon coating inhibits the side reaction between electrode and electrolyte as well as opens the reversible reaction path of the sulfur widely. It is worth noting that the ICE is gradually decreased with the increase in CDs, which might be caused by more irreversible capacity of insertion reaction in carbon materials and the alloying reaction of Sb. But it’s still pretty that the natural stibnite modified by CDs has infinite possibilities in the practical application of batteries. Note that the first discharge/charge curves of four samples exhibit different reaction platforms. During the initial discharge process, two apparent platforms at about 1.5 and 0.8 V are observed, which are associated with the conversion of Sb_2_S_3_ and the alloying reaction between Li^+^ and Sb. Thus, the discharge process can be validly divided into three stages: insertion reaction (3–1.5 V), conversion reaction (1.5–0.8 V), and alloying reaction (0.8–0.01 V). As for Sb_2_S_3_@0.5CDs, there are just insertion reaction (3–0.8 V) and alloying reaction (0.8–0.01 V).

Comparing with stibnite Sb_2_S_3_, Sb_2_S_3_@0.1CDs, and Sb_2_S_3_@0.3CDs anodes have excellent reversible conversion of sulfur in the charging process. The sulfur-doped carbon layer outside, as a multi-ply rampart, can extend the dissolution tunnels to bring down the loss rate of polysulfide and restrain the huge volume expansion as well. Besides, it is noted that all Sb_2_S_3_ have been reduced in Sb_2_S_3_@0.5CDs, no conversion reaction stage of sulfur is observed. In Fig. 5e, all platforms of Sb_2_S_3_@xCDs are maintained well in the charge–discharge cycles in stepwise current densities. In Fig. 5f, Sb_2_S_3_@0.3CDs electrode delivers the reversible capacity of 648.1 mA h g^−1^ at 0.1 A g^−1^ with capacity retention of 77% after 100 cycles, which is better than that of stibnite Sb_2_S_3_ (491.1 mAh g^−1^ after 100 cycles with the capacity retention of 69%). Due to the activation behavior caused by large particle size, the stibnite Sb_2_S_3_ anode has a little initial increase in capacity (< 20 cycles). The interesting composite architecture with a certain and appropriate amount of carbon matrix and rich active sites can render the Sb_2_S_3_@0.3CDs hybrid with stable cycling capacity. When the dosage of CDs is small, the physicochemical properties of the complex are more inclined to stibnite fundamentally, such as Sb_2_S_3_@0.1CDs. Clearly, Sb_2_S_3_@0.5CDs with superfluous CDs shows superior cycle stability (< 70 cycles). However, the irregular agglomeration of carbon has a negative effect on its persistent cycling performance that the capacity fading in the repeated charge/discharge reactions. In order to make clear the cause of capacity decline, discharge capacity contributions in three reaction stages at constant cycles are calculated as shown in Figs. 5g and S8. In the conversion reaction (1.5–0.8 V), the discharge capacity contribution of Sb_2_S_3_ is raised to 63.8% and then declines constantly which is well consistent with the variation of the capacity during the cycle, indicating that the activation process plays a prominent part in the conversion reaction. “Shuttling effect” of polysulfide and the poor reversibility of sulfur are the primary reasons for the continuous decrease in conversion reaction efficiency. As for Sb_2_S_3_@0.1CDs and Sb_2_S_3_@0.3CDs, irregular lamellar layers of sulfur-doped carbon, served as octopus tentacles, provide a strong attraction to prevent the loss of sulfur. Therefore, its capacity contribution rate of conversion reaction is relatively stable compared with stibnite Sb_2_S_3_. Obviously, the reversibility of the conversion reaction becomes better with the increase in carbon matrix and the partial reduction of stibnite. For example, the contribution rate of conversion reaction is always held at 58% in the Sb_2_S_3_@0.3CDs anode. There is no conversion reaction platform for Sb_2_S_3_@0.5CDs, suggesting that its capacity contribution is merely composed of insertion reaction and alloying reaction. What’s more, long-term cycling behaviors of the four samples at a higher current density of 0.5 A g^−1^ are exhibited in Fig. S9, and the residual capacity of Sb_2_S_3_@0.3CDs maintains 587.7 mAh g^−1^ with the retention of 74% after 200 loops, much higher than that of stibnite Sb_2_S_3_ (313.1 mAh g^−1^, 50%).

### Mechanism of Lithium Storage

**Fig. 6:**
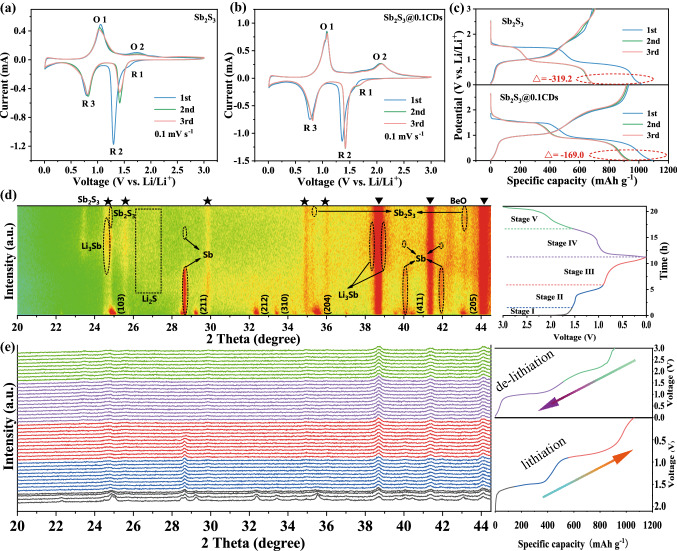
Mechanism analysis of the lithium storage process. CV curves at a scan rate of 0.1 mV s^−1^ of **a** Sb_2_S_3_ electrode and **b** Sb_2_S_3_@0.1CDs electrode. **c** GCD curves at 0.1 A g^−1^ of stibnite Sb_2_S_3_ and Sb_2_S_3_@0.1CDs electrodes. **d, e** In situ XRD patterns of Sb_2_S_3_@0.1CDs electrode with the corresponding GCD curves at 0.1 A g^−1^ during the first cycle

Normally, the initial discharge/charge profiles, cyclic voltammetry (CV) curves at a tiny scan rate and the in situ XRD should be applied conjunctively in exploring the lithium storage mechanism in the round [38]. The CV curves at 0.1 mV s^−1^ are shown in Figs. 6a–b and S10. There are three cathodic peaks at around 1.72, 1.45, and 0.80 V during the cathodic scan, which correspond to the insertion of Li^+^ into the Sb_2_S_3_/Sb_2_S_3_@xCDs host (as discussed below), the conversion of Sb_2_S_3_ into Li_2_S and metallic Sb, and the forming of Li_3_Sb through an alloying reaction between Sb and Li^+^, respectively. Compared to the results of the first and subsequent scans, there are slight shifts for the three reduction peaks (1.72 to 1.75 V, 1.45 to 1.49 V, 0.80 to 0.85 V), attributed to the formation of SEI, polymeric gel-like layer as well as the normal electrochemical alloying reaction, which is severer in Sb_2_S_3_@0.3CDs and Sb_2_S_3_@0.5CDs anodes. During the first anodic scan, a sharp peak at about 1.15 V is related to the dealloying process of Li_3_Sb, while the broad bulge at the range of 1.5–2.5 V is attributed to the formation of Sb_2_S_3_ through the conversion reaction between Li_2_S and Sb. Due to the structural collapse and the poor reversibility of sulfur, the oxidation peak (O2) of stibnite Sb_2_S_3_ is becoming lower gradually in the subsequent scans, showing negative reversibility. Except for stibnite Sb_2_S_3_, the 2^nd^ and 3rd CV curves of Sb_2_S_3_@xCDs nearly overlap, indicating that there are several advantages of low polarization, good stability, and high reversibility in Sb_2_S_3_@xCDs anodes. Apart from CV curves, discharge/charge curves also reflect the mechanism of lithium storage. In Fig. 6c, it could be seen that the capacity of initial discharge is up to 1024 mAh g^−1^, well over that of the second discharge. Meanwhile, the lengthy platform at 1.72 V during the 1st cycle is in sharp contrast to that of the 2nd cycle, suggesting that frequent side reactions and vast electrolyte decomposition take place in the conversion stage. Besides, the platform at 2.1 V nearly disappeared during the subsequent cycles, which is in perfect agreement with the CV curve of stibnite, inferring that the sulfide conversion reaction is inefficient and its reversible reaction is quite incomplete. On the contrary, once stibnite Sb_2_S_3_ is treated with CDs, the loss between the 1st and 2nd turns of discharge has a decreased amplitude. As displayed in Figs. 6c and S11, the capacity gap between two laps narrows to 147 mAh g^−1^ and it is mainly derived from alloying reaction. What’s more, this is an unprecedented improvement for the formation of Sb_2_S_3_ at 2.1 V, in terms of stability and reversibility. With an augment of metallic Sb in the composites, electrochemical alloying reaction becomes the key force for the first capacity increase due to the reduction of conversion reaction relied on Sb_2_S_3_ [39]. Through the comparison of four samples anodes, the high ICE of Sb_2_S_3_@xCDs mainly comes from two parts: large amounts of electrolyte invalidation in the first process of discharging and the inadequate conversion between Li_2_S and Sb during the charging process.

More importantly, in situ XRD is conducted to fully explore the phase transition and the lithium storage mechanism within the voltage range of 0.01–3.0 V (*vs*. Li^+^/Li). XRD patterns measured from 20° to 44.5° are displayed in Fig. 6d–e as well as the corresponding galvanostatic charge–discharge (GCD) curves. In the contour plot, red represents the maximum intensity of the XRD peaks, while green is the opposite. Notably, peaks located at 38.7°, 41.4°, and 44.1° all come from BeO that Be is used as the current collector for in situ cell test. In addition, some of the persistent peaks are derived from incomplete reactions of Sb_2_S_3_ which are marked in the picture. With the discharge process progressively going on, (103), (211), (212), (310), (204), (411) planes of Sb_2_S_3_ descend and disappear continuously, corresponding to the occurrence of Li^+^ intercalation into Sb_2_S_3_ host. As the discharge process proceeds from 1.55 to 0.85 V, the conversion of Sb_2_S_3_ begins punctually and is increased gradually. Concretely, three conspicuous peaks of metallic Sb (PDF No. 35-0732) appear at 28.7°, 40.1°, and 42.0° while the peak of Li_2_S (PDF No. 26-1188) is at around 26°. Upon discharging to 0.01 V, all peaks of Sb are decreased along with the emersion of three new peaks at 24.5°, 38.3°, and 39.1°, which suggests the formation of Li_3_Sb (PDF No. 04-0438). At the beginning of the charge process to 1.53 V, Li_3_Sb has gradually vanished, and the diffraction peak intensity of Sb is observed, implying that the dealloying reaction between Li_3_Sb and Sb. As it is further charged to 3 V, the peaks of Li_2_S are weak as well the low crystallinity phase of Sb_2_S_3_ reappears, which illustrates the partially reverse conversion reaction of Li_2_S and Sb into Sb_2_S_3_. Based on the aforementioned results, the delithiation/lithiation of Sb_2_S_3_@xCDs electrode is divided into five stages, as below:

Discharge

Stage I (above-1.55 V, insertion reaction)

1 $${\text{Sb}}_{{2}} {\text{S}}_{{3}} + {\text{ xLi}}^{ + } + {\text{ xe}}^{ - } \to {\text{ Li}}_{{\text{x}}} \left[ {{\text{ Sb}}_{{2}} {\text{S}}_{{3}} } \right]$$

Stage II (1.55–0.85 V, conversion reaction)

2 $${\text{Li}}_{{\text{x}}} \left[ {{\text{ Sb}}_{{2}} {\text{S}}_{{3}} } \right] \, + \, \left( {{6} - {\text{x}}} \right){\text{ Li}}^{ + } + \, \left( {{6} - {\text{x}}} \right){\text{ e}}^{ - } \to {\text{ 2Sb }} + {\text{ 3Li}}_{{2}} {\text{S}}$$

Stage III (0.85–0.01 V, alloying reaction)

3 $${\text{Sb }} + {\text{ 3Li}}^{ + } + {\text{ 3e}}^{ - } \to {\text{ Li}}_{{3}} {\text{Sb}}$$

Charge

Stage IV (0.01–1.53 V, dealloying)

4 $${\text{Li}}_{{3}} {\text{Sb }} \to {\text{ Sb }} + {\text{ 3Li}}^{ + } + {\text{ 3e}}^{ - }$$

Stage V (1.53–3.0 V, reversed conversion)

5 $${\text{3Li}}_{{2}} {\text{S }} + {\text{ 2Sb }} \to {\text{ Sb}}_{{2}} {\text{S}}_{{3}} + {\text{ 6Li}}^{ + } + {\text{ 6e}}^{ - } .$$

### Analysis of the High Initial Coulombic Efficiency

**Fig. 7:**
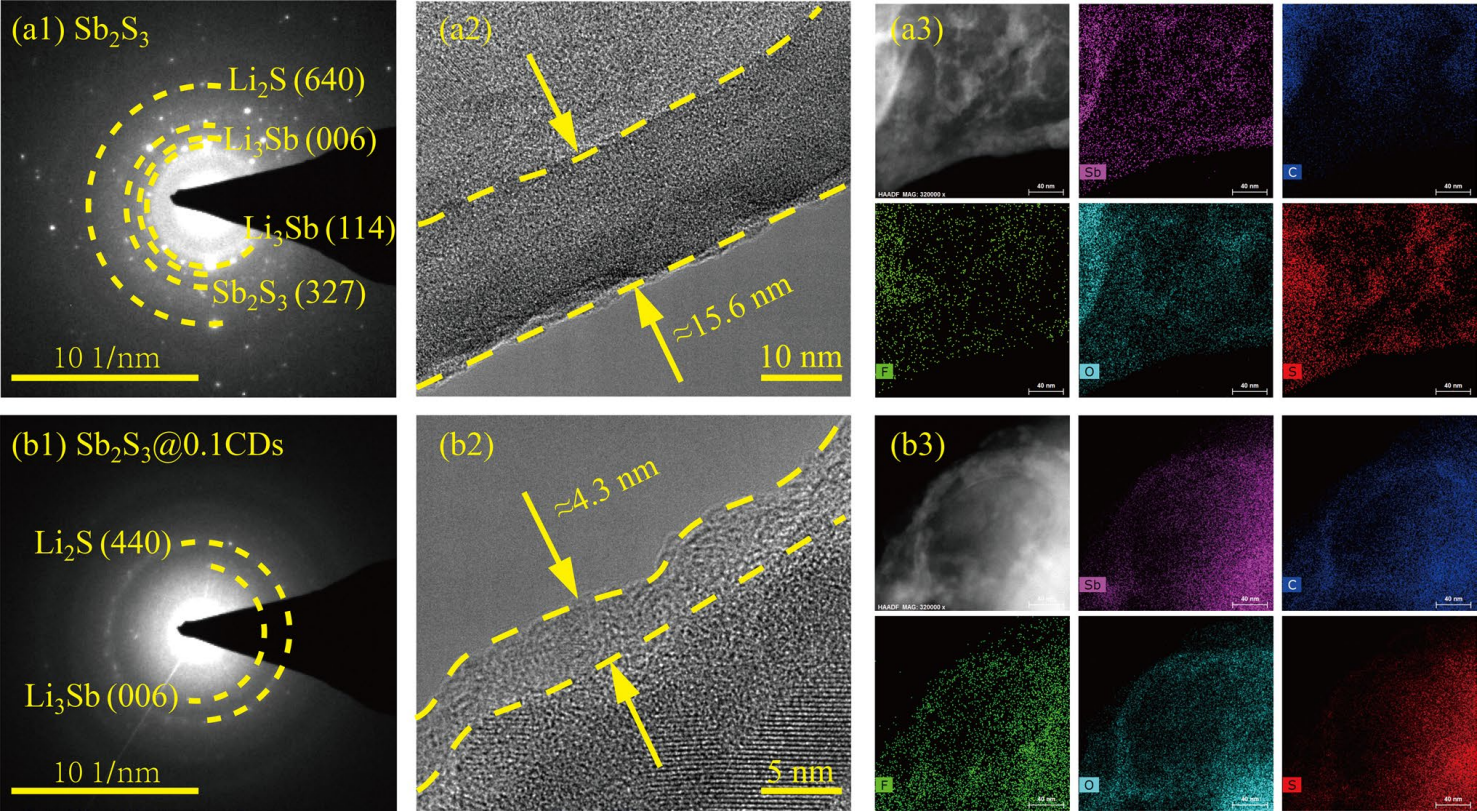
SEI characterization. SAED patterns of (**a1**) stibnite Sb_2_S_3_ and (**b1**) Sb_2_S_3_@0.1CDs. HRTEM of SEI formed on (**a2**) stibnite Sb_2_S_3_ and (**b2**) Sb_2_S_3_@0.1CDs. The element mapping images of (**a3**) stibnite Sb_2_S_3_ and (**b3**) Sb_2_S_3_@0.1CDs. All states are discharged to 0.01 V in the first cycle

For reason of answering to the riddle of high ICE, the structure and components of SEI adhered to the surface of Sb_2_S_3_@xCDs are measured concretely via HRTEM and in-depth XPS. As shown in Figs. 7 and S12, different phases are clearly observed when the electrodes are discharged to 0.01 V. There are four distinct diffraction rings in the SAED pattern, well indexing to Li_2_S (640), Li_3_Sb (006) (114), and Sb_2_S_3_ (327), respectively, which indicates the inexhaustive reaction of Sb_2_S_3_. And the presence of weaker diffraction rings of Li_2_S (440) and Li_3_Sb (006) certifies the successful conversion reaction and alloying reaction objectively in Sb_2_S_3_@xCDs electrodes. After the first discharge process, the reaction of stibnite Sb_2_S_3_ modified by CDs is more thorough. Besides, it is apparent in Fig. 7(a2) that a thick and non-uniform SEI layer is about 7.4 nm in natural stibnite electrode. On the contrary, a thin and uniform SEI layer (≈4.3 nm) is formed on the surface of Sb_2_S_3_@0.1CDs electrode (Fig. 7(b2)) as well as that of Sb_2_S_3_@0.3CDs (≈5.1 nm) (Fig. S12b). Notably, the formation of an ultrathin and stable SEI film can suppress the decomposition of the electrolyte, cut down the undesirable reaction and boost Li^+^ ions diffusion through the surface of active components [40]. In addition, the results of EDS elemental mapping (Figs. 7(a3–b3) and S12(c1–c3)) demonstrate the distribution of Sb, C, F, O, and S, verifying that the generated substances in SEI layer are evenly distributed.

**Fig. 8:**
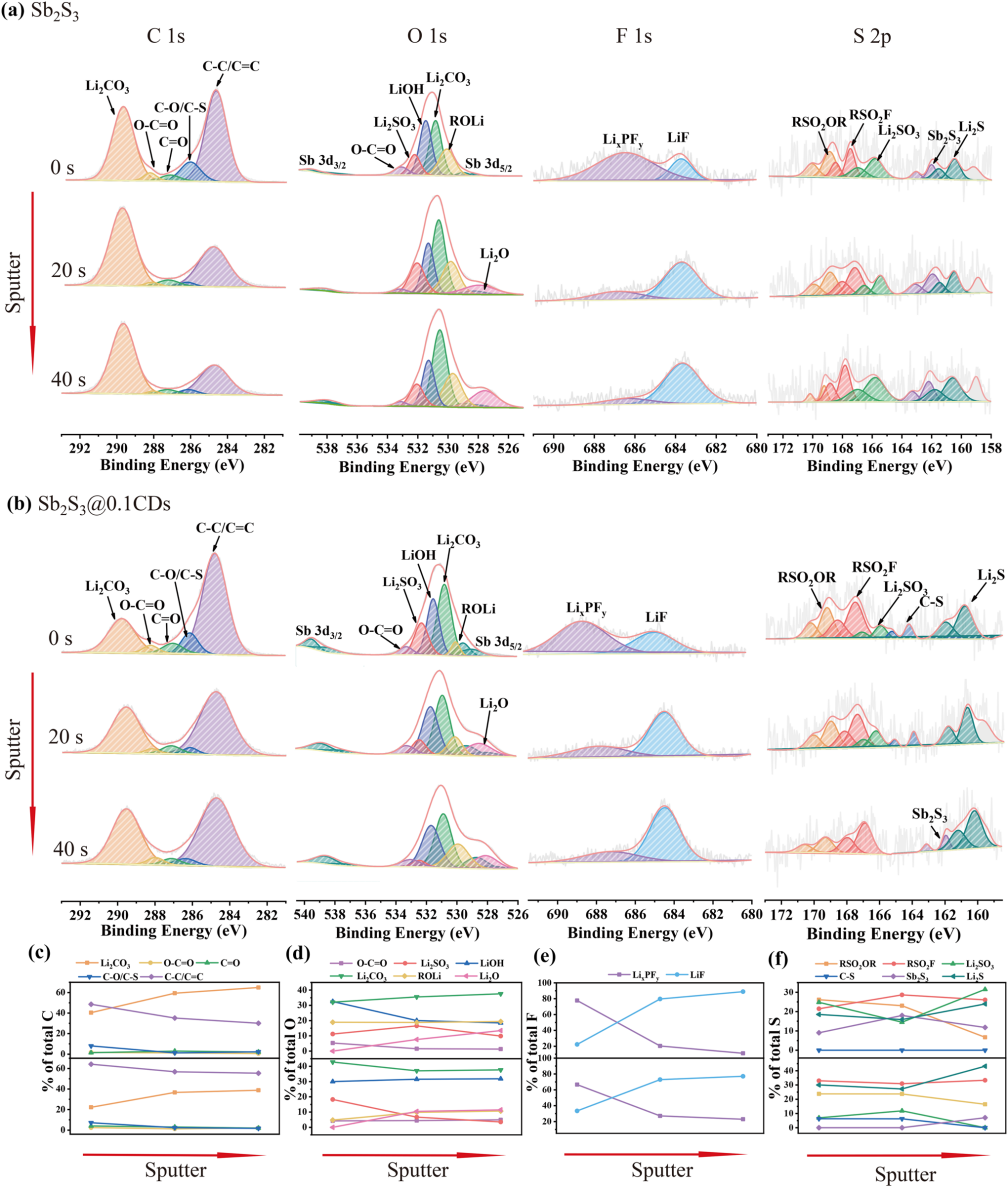
High-resolution XPS spectrum of **a** stibnite Sb_2_S_3_ and **b** Sb_2_S_3_@0.1CDs with sputtering time for 0, 20, and 40 s, respectively, and the corresponding proportions of primary existence forms of **c** C, **d** O, **e** F and **f** S elements. All states are discharging to 0.01 V in the first cycle

**Fig. 9:**
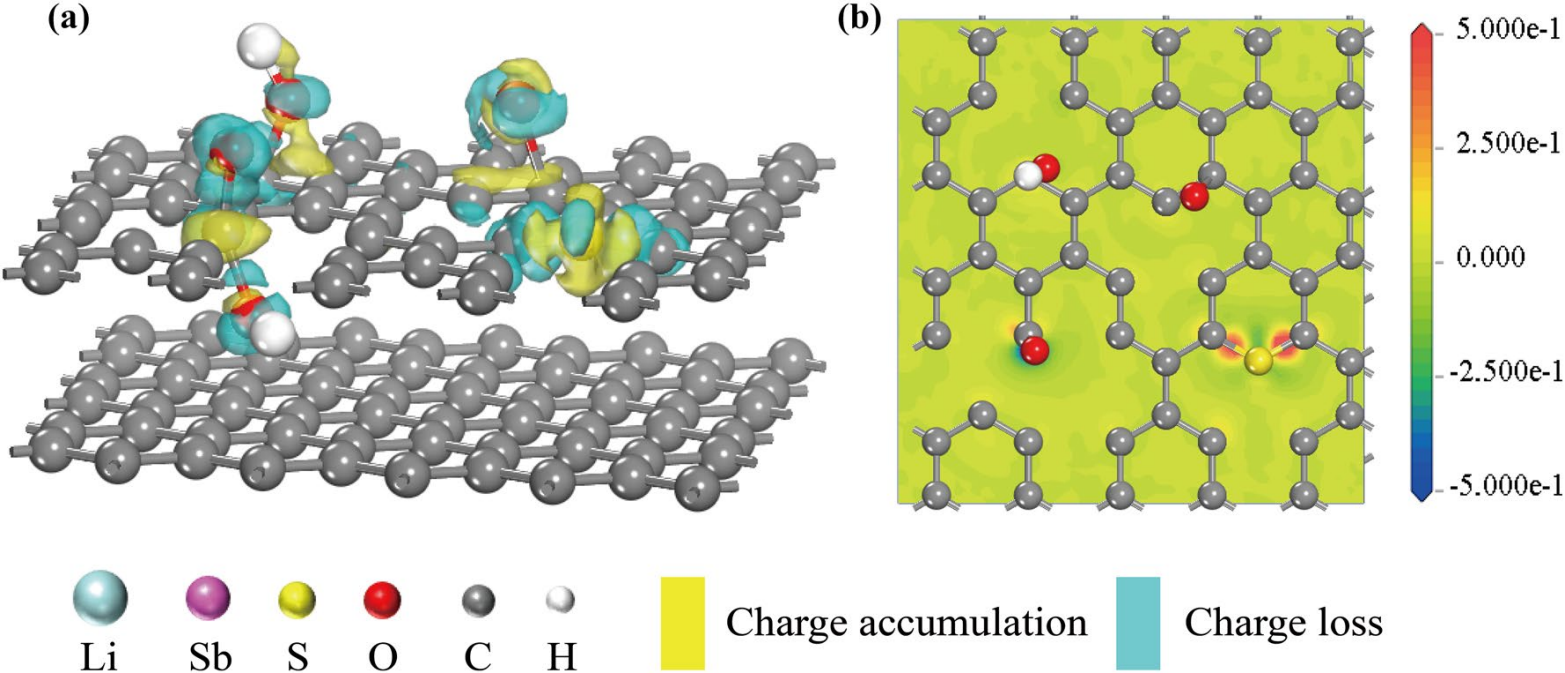
The charge density of the oxygen-containing functional groups on the carbon matrix

According to TEM results, the chemical composition of SEI is measured via XPS with an Ar^+^ sputtering in depth [41]. Figure 8a, b shows the changes of C 1*s*, O 1*s*, F 1*s*, and S 2*p* in the SEI films of Sb_2_S_3_ and Sb_2_S_3_@0.1CDs, respectively, with the increased sputtering time. Meanwhile, the quantitative results of the changes in the composition of these four elements (C, O, F, and S) over time are shown in Fig. 8c–f, respectively. The decreasing content of C suggests that outer SEI is mainly composed of organic components [42]. From the O 1*s* spectrum, it is found that the composition of inorganic materials is truly complex. As the etch deepens, there are obvious changes in contents of Li_2_SO_3_ and Li_2_O with the former being smaller and the latter increasing. Meanwhile, LiF replaces Li_x_PF_y_ as the main part of F 1*s* spectra after sputtering 20 s. As an electron insulator, LiF possesses a relatively high ionic conductivity (≈10^–8^ S cm^−1^) while Li_2_CO_3_ has a relatively poor ionic conductivity known as an electron conductor [43]. The heterogeneous structure between them has an excellent synergetic effect that can not only inhibit the decomposition of electrolyte solution, but also assist the transport of Li^+^ ions [44]. Moreover, LiF-rich SEI yields great advantages in regulating the homogeneous deposition of Li^+^ ions flux and preventing the growth of dendrites [45]. The S 2*p* spectrum displays the complex diversity of sulfur element conversion, including organics such as RSO_2_OR, RSO_2_F, inorganic Li_2_SO_3_ and Li_2_S [46]. In stibnite Sb_2_S_3_ anode, Li_2_SO_3_ is the main component of inorganic SEI throughout whole deepening sputtering process, which means an irreversible loss of sulfur. For comparison, with sputtering time going on, the content of Li_2_SO_3_ is gradually tapered off and eventually disappears. This stark difference can be probably attributed to the existence of C-S bonds in a unique environment within abundant oxygen-containing functional groups. Besides, the EIS analysis is used to explore R_SEI_ after the first cycle at 0.1 A g^−1^ in Fig. S13. There are two semicircles in the high-frequency region and a straight line in the low-frequency region in the Nyquist plots, which is corresponding to the resistances of SEI layers (*R*_SEI_), charge-transfer resistances (*R*_ct_) at interfaces, and the Warburg impendence (*Z*_w_) during the processes of Li^+^ ions diffusion, respectively [26]. Obviously, the first semicircle of Sb_2_S_3_ is larger than other three samples which is aligned with the higher *R*_SEI_ of Sb_2_S_3_. In order to test this bold conjecture, the DFT calculations are employed to figure out the difference between C–S bonds and Sb–S bonds from an atomic insight. Firstly, the bond length of C–S is only 1.469 Å, almost half that of the Sb–S bond (2.681 Å). In addition, the bond energy of C–S (152.75 kJ mol^−1^) is greatly higher than Sb–C bond energy (96.853 kJ mol^−1^). Therefore, it is proposed that C–S bonds are steadier thanks to their shorter bond length and stronger bond energy, making them harder to form adverse sulfite. Furthermore, a well-defined model of charge density around oxygen-containing functional groups is displayed in Fig. 9a, mostly overlapping of charge accumulation (yellow) and charge depletion (cyan). It is clear seen that the electrons of O atoms obviously converge toward S atoms, spontaneously forming a high local charge density named “charge sea.” Meanwhile, the corresponding ichnography is depicted in Fig. 9b, blue and red represent the lowest and highest relative intensity of charge density, respectively. As described in the structural model, the charge is concentrated around the C-S bond. In a word, the large amount of oxygen-containing functional groups from carbon matrix derived from CDs, such as C–O, C=O, and O–C=O [19], creates a charge bias which directly affects the C–S bonds, making the C–S bonds more stable and thus acting as a sulfite suppressor. In addition, owing to the fact that carbon matrix with C–S bond is coated on the outer Sb_2_S_3_, which avoids direct contact between Sb_2_S_3_ and electrolyte. Under the combined action of these two aspects, the irreversible conversion reaction of sulfur to sulfate is inhibited.

### Dynamic Analysis

It is necessary for Sb_2_S_3_@xCDs electrodes to figure out the root of fast charging and kinetics analysis, combined with experimental characterizations and theoretical calculations. Based on the previous reports [47], the kinetic behaviors can be explored by the following equations in detail.

6 $$I_{p} = \, 2.69 \times 10^{5} n^{3/2} AD^{1/2} v^{1/2} C_{Li + }$$

7 $$i \, = \, a^{v} b$$

8 $$i \, = \, k_{1} v \, + \, k_{2} v^{0.5} .$$

**Fig. 10:**
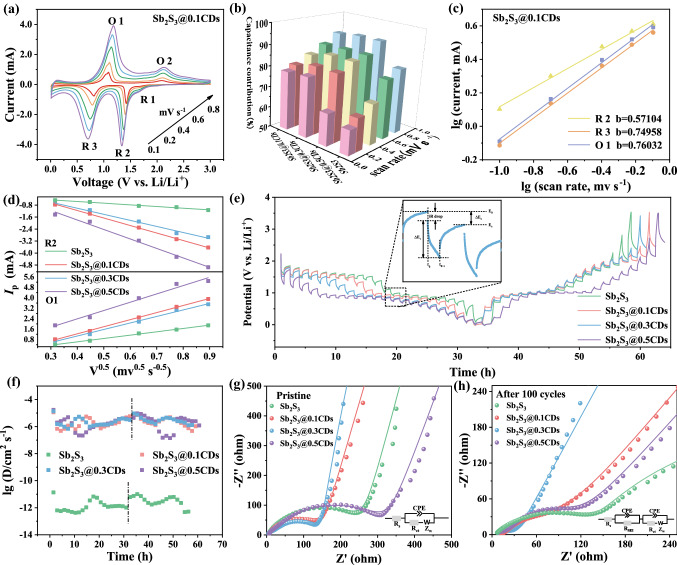
Electrochemical kinetics mechanism. **a** CV curves of Sb_2_S_3_@0.1CDs electrode at different scan rates. **b** Capacitive contribution of stibnite Sb_2_S_3_, Sb_2_S_3_@0.1CDs, Sb_2_S_3_@0.3CDs, and Sb_2_S_3_@0.5CDs at various scan rates. **c** Linear relations between log(v) and log(i) at peak currents corresponding to the CV curves of Sb_2_S_3_@0.1CDs electrode. **d** Linear relations between Ip and log(i) of stibnite Sb_2_S_3_ and Sb_2_S_3_@xCDs. **e** GITT potential profile of four samples and **f** the relationship between diffusion coefficient of Li^+^ ions and time. Electron-transfer character of **g** pristine and **h** cycled cells is performed by the EIS from 100 to 0.01 Hz

Firstly, the specific contributions of surface pseudocapacitance, as a significant parameter of rate performance, are calculated by CV profiles (Figs. 10a and S14) at stepwise scan rates. In Fig. 10b, the capacitive percentages of Sb_2_S_3_@xCDs electrodes are all much higher than that of stibnite Sb_2_S_3_. Meanwhile, with the increasing of scan rates, the capacitive behavior is obviously improved, which offers corresponding fast kinetics to promote the high rate of the Sb_2_S_3_@xCDs composites. As an important kinetic parameter, the *b*-value is used to distinguish the electrochemical rate control process and the ion-storage capacity controlling step [9]. The *b*-value near 1.0 stands for the surface-controlled behaviors whereas it closed to 0.5 is dominatingly determined by the diffusion process. The linear fitting relationships between log (*v*) and log (*i*) of four samples are displayed in Figs. 10c and S15. It reveals that surface-controlled behaviors are the major Li^+^ ions storage mechanism for stibnite Sb_2_S_3_ and Sb_2_S_3_@0.5CDs while the ion-storage capacity of Sb_2_S_3_@0.1CDs and Sb_2_S_3_@0.3CDs come from the synergetic effect of diffusion behaviors and surface-controlled behaviors [48]. The difference might be attributed to the larger and denser particle size and smaller specific surface area of Sb_2_S_3_@0.5CDs as well as the more serious agglomeration. As shown in Fig. 10d, the as-calculated slopes of four samples are measured to compare the corresponding Li^+^ ions migration coefficient (*D*_Li+_). It is worth noting that slops are gradually growing larger with the increased content of CDs in composites, suggesting that the migration rates of Li^+^ ions are advanced greatly. The galvanostatic intermittent titration (GITT) technique is also utilized to figure up D_Li+_. As depicted in Fig. 10e, the curves of four samples possess a semblable diffusion trend, equal to similar ion diffusion behaviors. Figures 10f and S16 show the relationship between time/voltage and the values of *D*_Li+_. Except for stibnite Sb_2_S_3_ anodes, all others show extraordinary *D*_Li+_ in the processes of lithiation and delithiation, which is attributed to the efficient conduction for charge transport rooted in the tight connection between carbon matrix and Sb_2_S_3_/Sb as well as the surface electron densities of stable Sb–O–C bonds. It can be observed in Fig. 10g that the Nyquist plots consist of a semicircle in high-frequency region and a slop line at low frequency, which is referred to the charge-transfer resistances (*R*_ct_) between the electrodes and electrolyte and the Warburg impendence (Z_w_) of lithium diffusion process. In particular, the *R*_ct_ of Sb_2_S_3_@0.3CDs (167.9 Ω) is much lower than that of stibnite Sb_2_S_3_ (211.3 Ω) in Table S1, indicating that the conductivity and charge transfer are largely improved by the Sb–O–C bonds and cross-linking carbon skeleton. Meanwhile, the slope of Sb_2_S_3_@0.3CDs is minimum, suggesting the least diffusion resistance. And after cycles, the lower *R*_ct_ manifests that the charge transport kinetics is promoted, probably because of structural integrity, and a lower energy barrier at interface due to a compact and stable SEI layer.

**Fig. 11:**
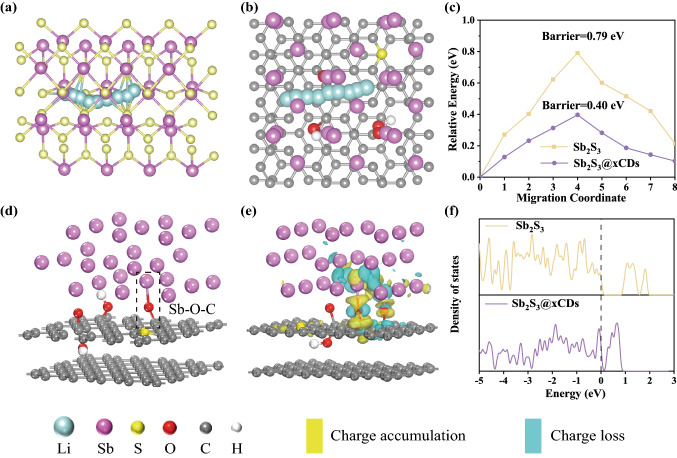
DFT calculations. Li^+^-ion migration pathway of **a** stibnite Sb_2_S_3_ and **b** Sb_2_S_3_@0.1CDs hybrids in the front view. **c** Comparison of Li^+^-ion diffusion energy barrier between stibnite Sb_2_S_3_ and Sb_2_S_3_@xCDs. **d** Structure of the Sb–O–C bond. **e** Charge density difference plot of Sb_2_S_3_@xCDs. The light blue and yellow areas indicate the electron loss and accumulation, respectively. **f** Density of states (DOS) profiles of stibnite Sb_2_S_3_ and Sb_2_S_3_@xCDs

DFT calculations are always utilized to investigate the affinity and reaction of Li with Sb_2_S_3_ and seek for the interactions of oxygen-containing functional groups on the carbon matrix at the atomic level [49]. Based on the above-mentioned physicochemical properties, the specific DFT models of Sb_2_S_3_, Sb, and Sb_2_S_3_@0.1CDs hybrids are built in Fig. S17. Based on the first-principles calculations, the electronic structure, diffusion barrier, and electronic conductivity of stibnite and Sb_2_S_3_@0.1CDs composites were acquired, which drastically reveals how the heterogeneous interface of Sb_2_S_3_@0.1CDs affects Li^+^ ions transport [50]. Firstly, the Li^+^-ion diffusion pathways of stibnite Sb_2_S_3_ and Sb_2_S_3_@0.1CDs from three different perspectives are shown in Figs. 11a–b and S18. Then, the corresponding diffusion energy barriers of Sb_2_S_3_ and Sb_2_S_3_@0.1CDs with the constant change of migration path are presented in Fig. 11c. Notably, the energy barrier of Sb_2_S_3_ is 0.79 eV, nearly twice as high as that of Sb_2_S_3_@0.1CDs (0.4 eV), indicating that the diffusion resistance of Li^+^ ions is decreased through the heterointerface of Sb_2_S_3_@0.1CDs concatenated by Sb–O–C, and further reinforce the conductivity of ions [51]. Moreover, the calculated density of state (DOS) displays a broad bandgap (≈0.59 eV) of the stibnite Sb_2_S_3_ compared to the Sb_2_S_3_@0.1CDs system with zero bandgap near the Fermi level in Fig. 11f. It is important for the narrow bandgap to enhance electronic conductivity, verifying greatly efficient electron transport after introduction of heterointerface with conductive carbon matrix [26]. Theoretically, Sb–O–C bonds in Fig. 11d are organized by the oxygen-containing functional groups attached on CDs which has been proved in the results of XPS and Raman. As shown in Fig. 11e, the charge density difference (CDD) is mapped to explore the behavior of electronic charge transfer across the heterointerfaces between Sb, O, and C atoms. Interestingly, the overlapping of charge accumulation and charge depletion reveals the formation of Sb–O–C bond, facilitating the fast charge transfer of Sb_2_S_3_@0.1CDs as well as stabilizing the slip of the dislocation.

In addition, stibnite Sb_2_S_3_ and Sb_2_S_3_@xCDs were also used as electrodes for SIBs. Like LIBs, the rate performance of Sb_2_S_3_@xCDs is greatly improved, especially at high current density, and the ICE can also reach ~ 83% in Fig. S19. GCD curves at 0.1 A g^−1^ of stibnite Sb_2_S_3_ and Sb_2_S_3_@xCDs in Fig. S20 also exhibit the increasing reversibility of sulfur and the decreasing loss of electrolyte in the first discharge. The test results of electrochemical kinetics are displayed in Figs. S21–S23.

## Conclusion

In summary, a unique structure of Sb_2_S_3_@Sb@C with strong interfacial chemical bond was designed to promote the lithium/sodium storage performance of Sb_2_S_3_ by a facile one-step heat treatment of low-cost natural stibnite and carbon dots with oxygen-containing functional groups. The detailed evolution mechanism was studied systematically and proposing the local oxidation—partial reduction—deep coupling mechanism, which can be extended to build other metal sulfides/carbon composite. In comparison with raw stibnite, the obtained Sb_2_S_3_-based composite exhibited superior electrochemical properties, especially the initial Coulombic efficiency, which is as high as 85%, much higher than that of the reported Sb_2_S_3_ materials. It was demonstrated that the formation of C–S bond in the CDs-derived carbon matrix is the key for the high ICE, which can inhibit the irreversible conversion of sulfur to sulfite, thus reducing the irreversible loss of sulfur and establishing thin, compact, and stable SEI layers. And the Sb–O–C interaction at the interface can significantly enhance its inherent electronic conductivity and restrain the slip of the dislocation which is confirmed roundly by experiment results and DFT calculations. Additionally, the introduction of carbon matrix and in situ formed metallic Sb can also enhance the structure stability and electronic conductivity, leading to improved cycle stability and rate performance. This work not only provides an original insight to improve the ICE of metal sulfides in their applications of batteries, but also offers a new perspective for natural minerals to be used as energy storage materials.

## Supplementary Information

Below is the link to the electronic supplementary material.

Supplementary file1 (PDF 3389 kb)
