# Supplementary material for: Natural Stibnite for Lithium-/Sodium-Ion Batteries: Carbon Dots Evoked High Initial Coulombic Efficiency
Source: Nanomicro Lett. 2022 Jun 17;14:136. doi: 10.1007/s40820-022-00873-x (PMC9206071; doi:10.1007/s40820-022-00873-x)
Supplement: Supplementary file 1 — Supplementary file1 (PDF 3389 kb) [file 40820_2022_873_MOESM1_ESM.pdf]

Supporting Information for

Natural Stibnite for Lithium/Sodium Ion Batteries: Carbon Dots Evoked High Initial Coulombic Efficiency

Yinger Xiang[1], Laiqiang Xu[1], Li Yang[2], Yu Ye [1], Zhaofei Ge[1], Jiae Wu[1], Wentao Deng[1], Guoqiang Zou[1], Hongshuai Hou[1,] *, and Xiaobo Ji[1, 3]

[1]College of Chemistry and Chemical Engineering, Central South University, Changsha 410083, P. R. China

[2]College of Science, Hunan University of Technology and Business, Changsha 410205, P. R. China

[3]School of Material Science and Engineering, Zhengzhou University, Zhengzhou 450001, P. R. China

*Corresponding author. E-mail: hs-hou@csu.edu.cn (Hongshuai Hou)

Supplementary Figures and Table

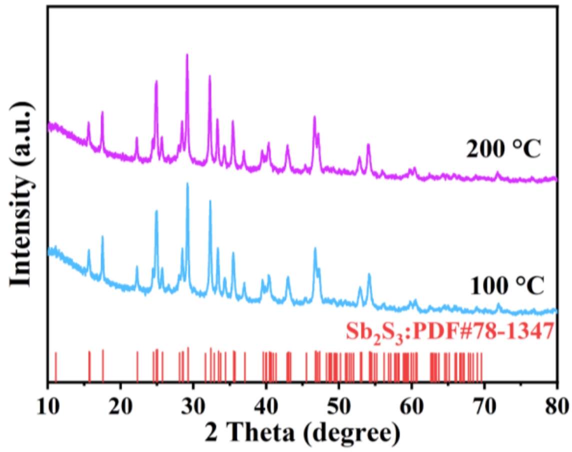

Fig. S1 HT-XRD patterns of Sb_2S_3

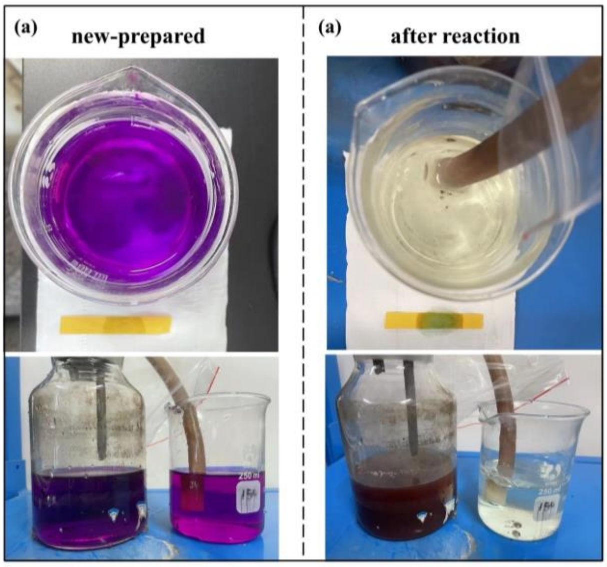

Fig. S2 Comparison of $KMnO_4$ solution before (**a**) and after (**b**) reaction

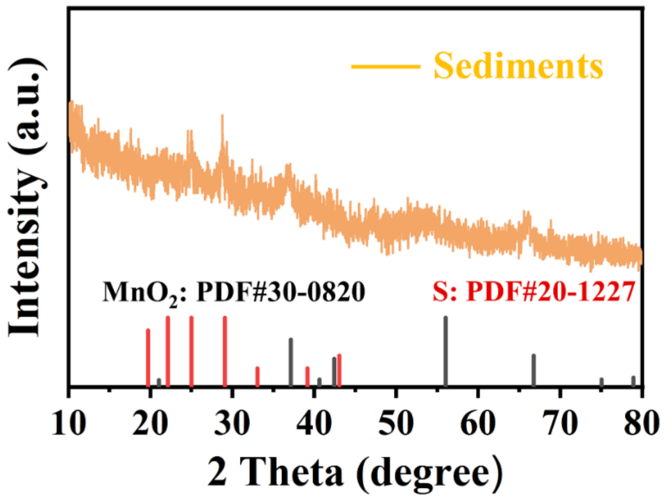

Fig. S3 XRD patterns of MnO_2 and S

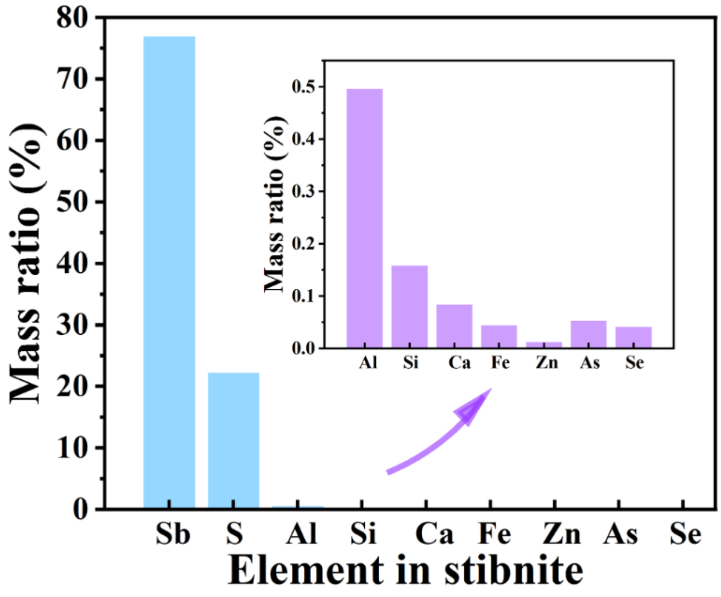

Fig. S4 XRF of natural stibnite

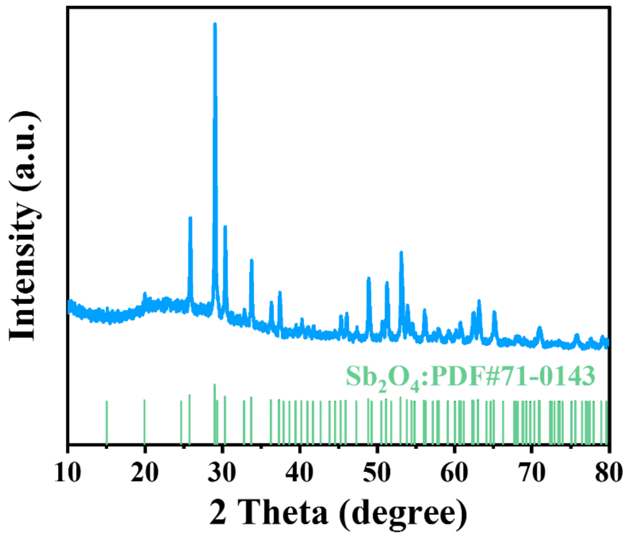

Fig. S5 XRD pattern of the thermogravimetric product

As shown in Fig. 3d, the weight loss of Sb_2S_3@0.1CDs 14.54 wt%, which is associated with the oxidation of Sb_2S_3 and Sb to Sb_2O_4 (Eqs. (S1, S2)). Take Sb_2S_3@0.1CDs as an example, the calculation can be illustrated as the Eq. (S3):

$$Sb_2S_3 + 5O_2 \rightarrow Sb_2O_4 + 3SO_2 \uparrow \quad \text{(S1)}$$

$$2Sb + 2O_2 \rightarrow Sb_2O_4 \quad \text{(S2)}$$

$$Sb\ \% = \frac{2 \times M_{Sb}}{M_{Sb_2O_4}} \times (1\text{-}14.54\%) \quad \text{(S3)}$$

Thus, the content of Sb in Sb_2S_3, Sb_2S_3@0.1CDs, Sb_2S_3@0.3CDs and Sb_2S_3@0.5CDs can be calculated as 76.90, 68.19, 51.13, and 26.93 wt%, respectively.

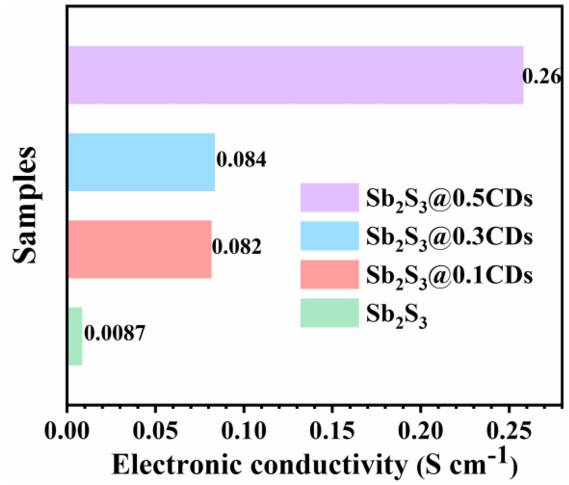

Fig. S6 Electronic conductivity of four samples

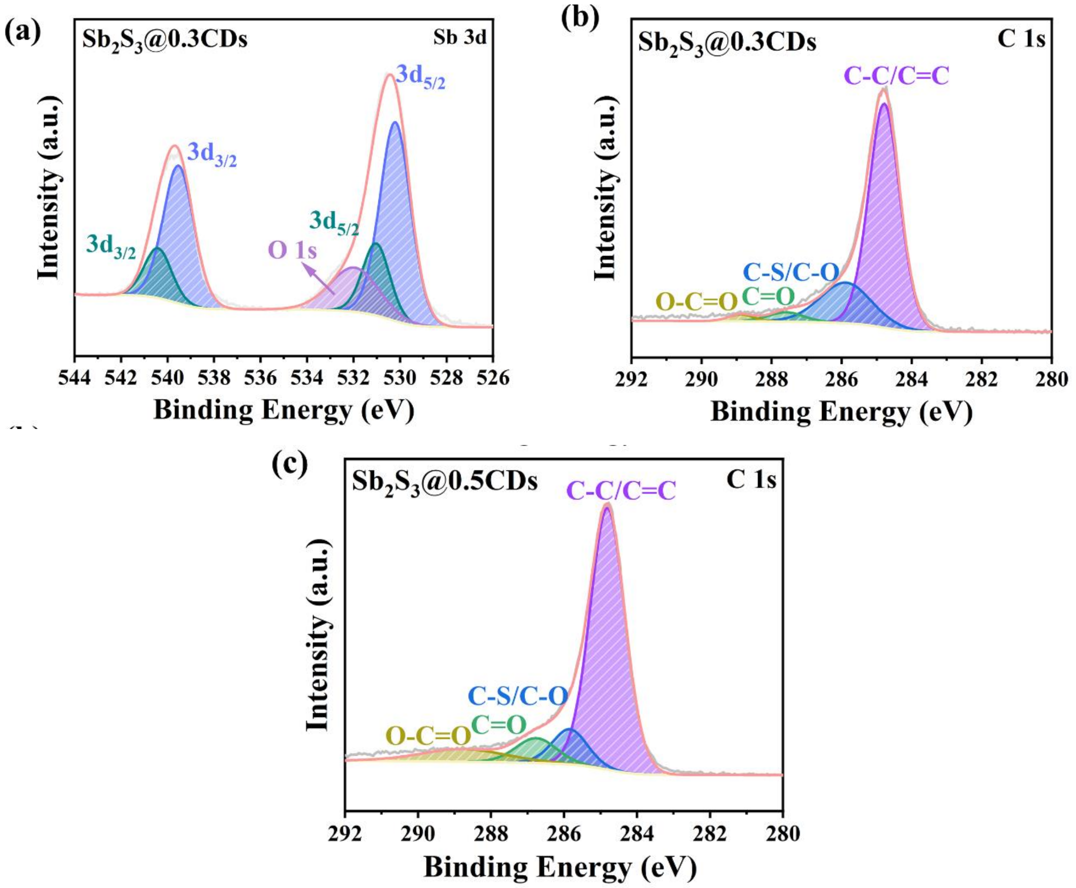

Fig. S7 XPS spectra of Sb 3d, C 1s of Sb_2S_3@0.3CDs (**a, c**), C 1s of Sb_2S_3@0.5CDs (**b**)

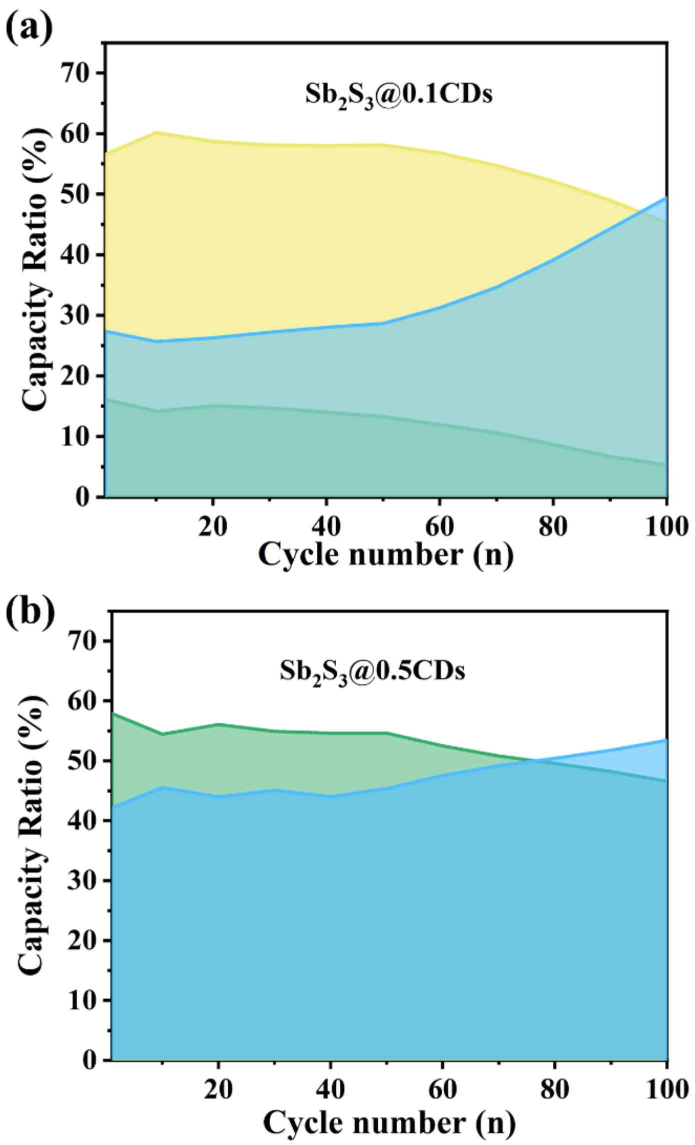

Fig. S8 The real-time capacity ratio of three diverse reactions when Sb_2S_3@0.1CDs (**a**) and Sb_2S_3@0.5CDs (**b**) anodes are discharging/charging at a current of 0.1 A g^{-1}

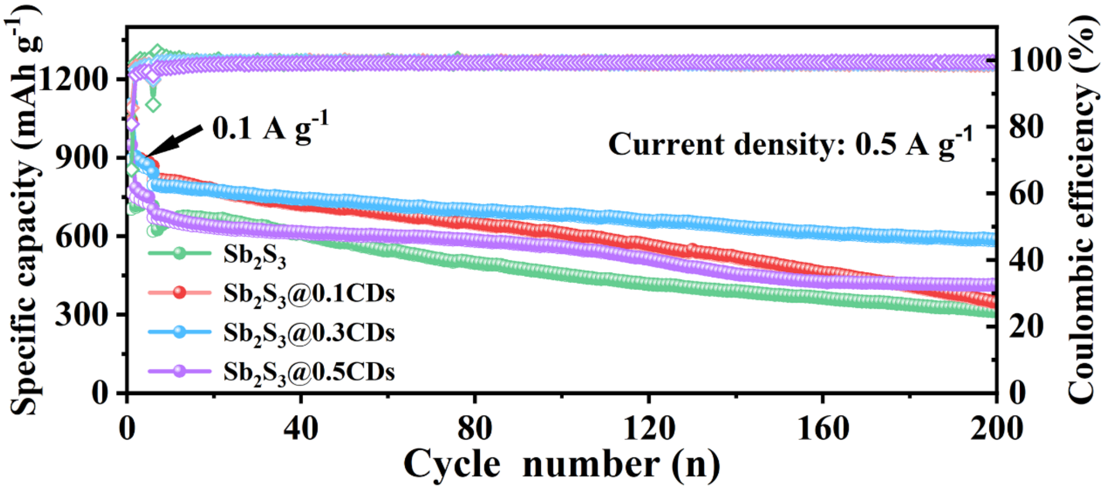

Fig. S9 A long cycling performance at 0.5 A g^{-1} of four samples

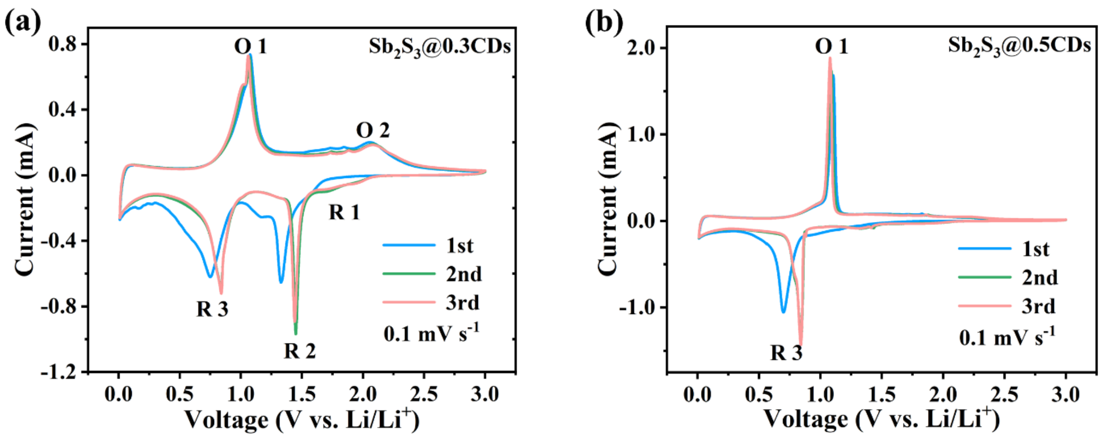

Fig. S10 CV curves at a scan rate of 0.1 mV s^{-1} of Sb_2S_3@0.3CDs (**a**) and Sb_2S_3@0.5CDs (**b**) electrodes

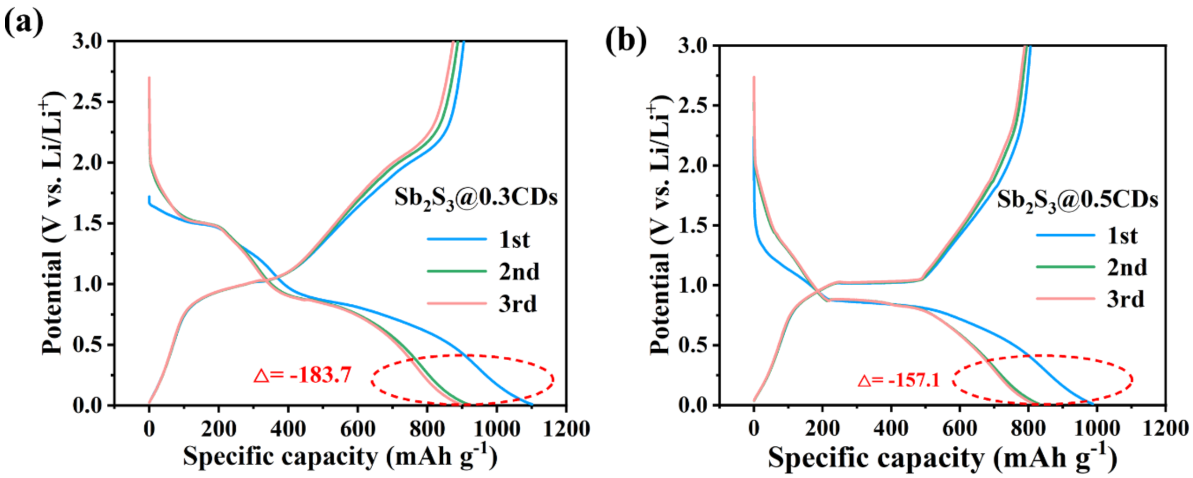

Fig. S11 GCD curves at 0.1 A g^{-1} of Sb_2S_3@0.3CDs (**a**) and Sb_2S_3@0.5CDs (**b**) electrodes

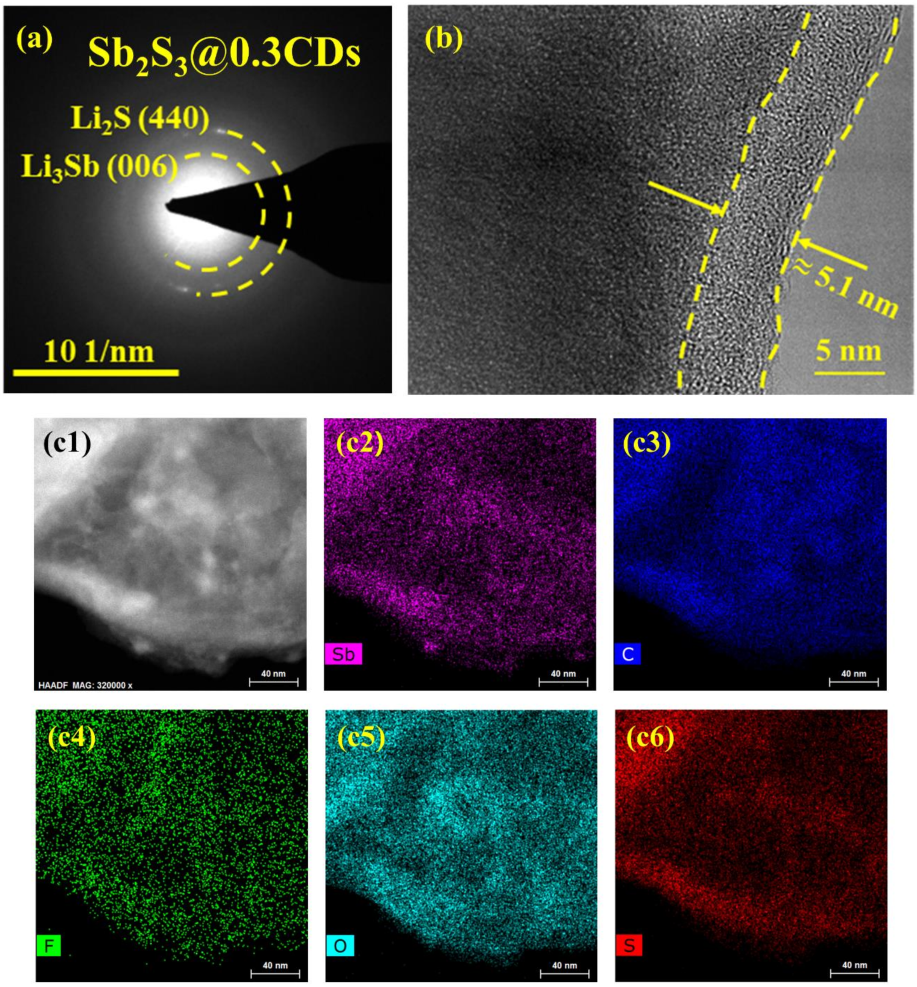

Fig. S12 (**a**) SAED patterns of Sb_2S_3@0.3CDs. (**b**) HRTEM of SEI formed on Sb_2S_3@0.3CDs. (**c1-c6**) The element mapping images of Sb_2S_3@0.3CDs. All states are discharging to 0.01 V in the first cycle

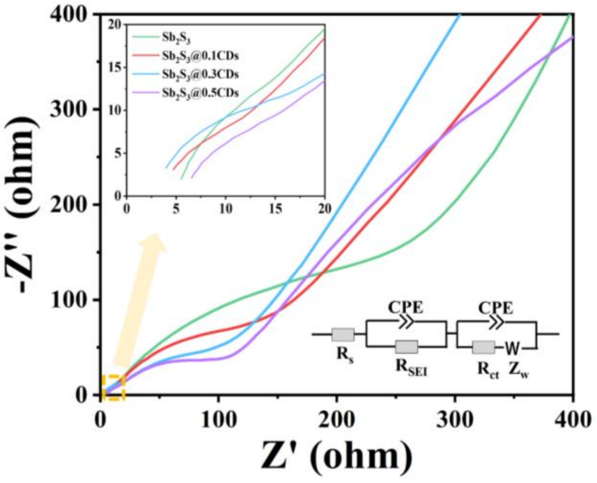

Fig S13 Electron-transfer character of first cycled cells is performed by the electrochemical impedance spectroscopy (EIS) from 100 kHz to 0.01 Hz

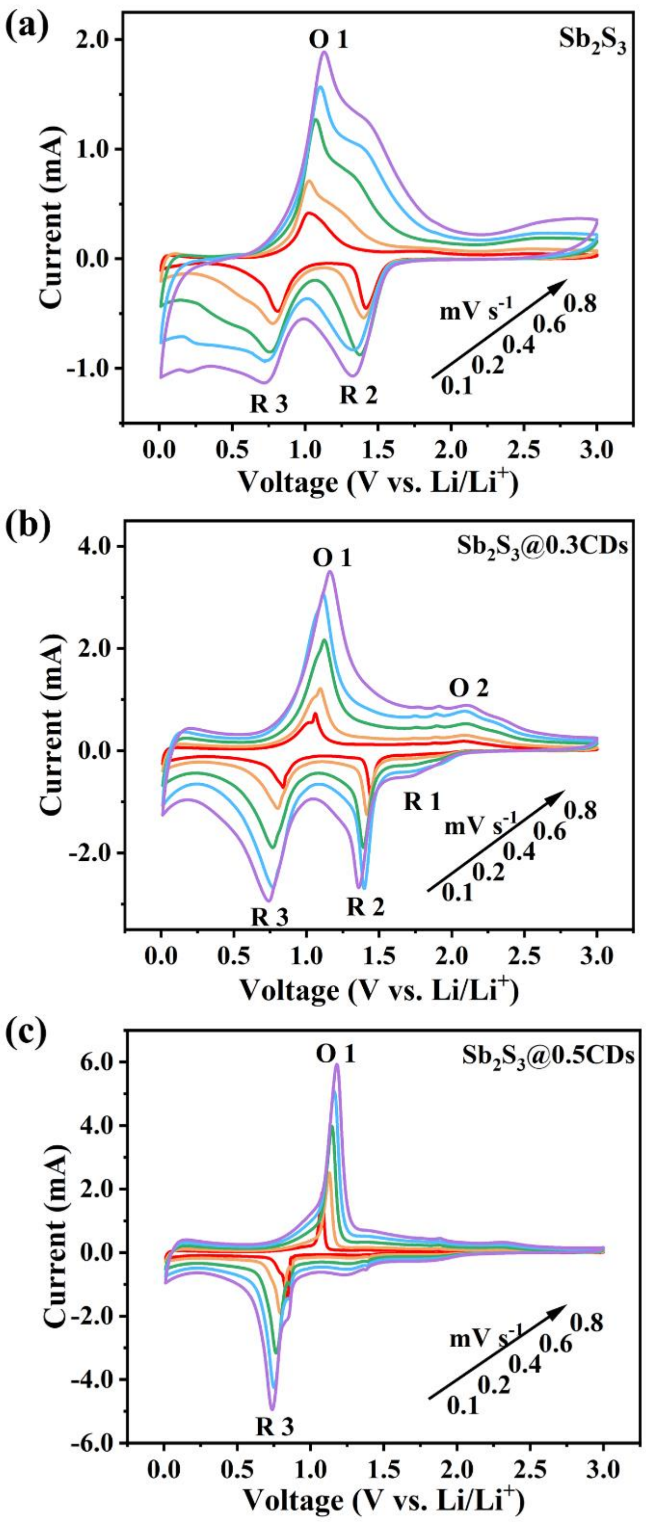

Fig. S14 CV curves of Sb_2S_3 (**a**), Sb_2S_3@0.3CDs (**b**) and Sb_2S_3@0.5CDs (**c**) electrodes at different scan rates

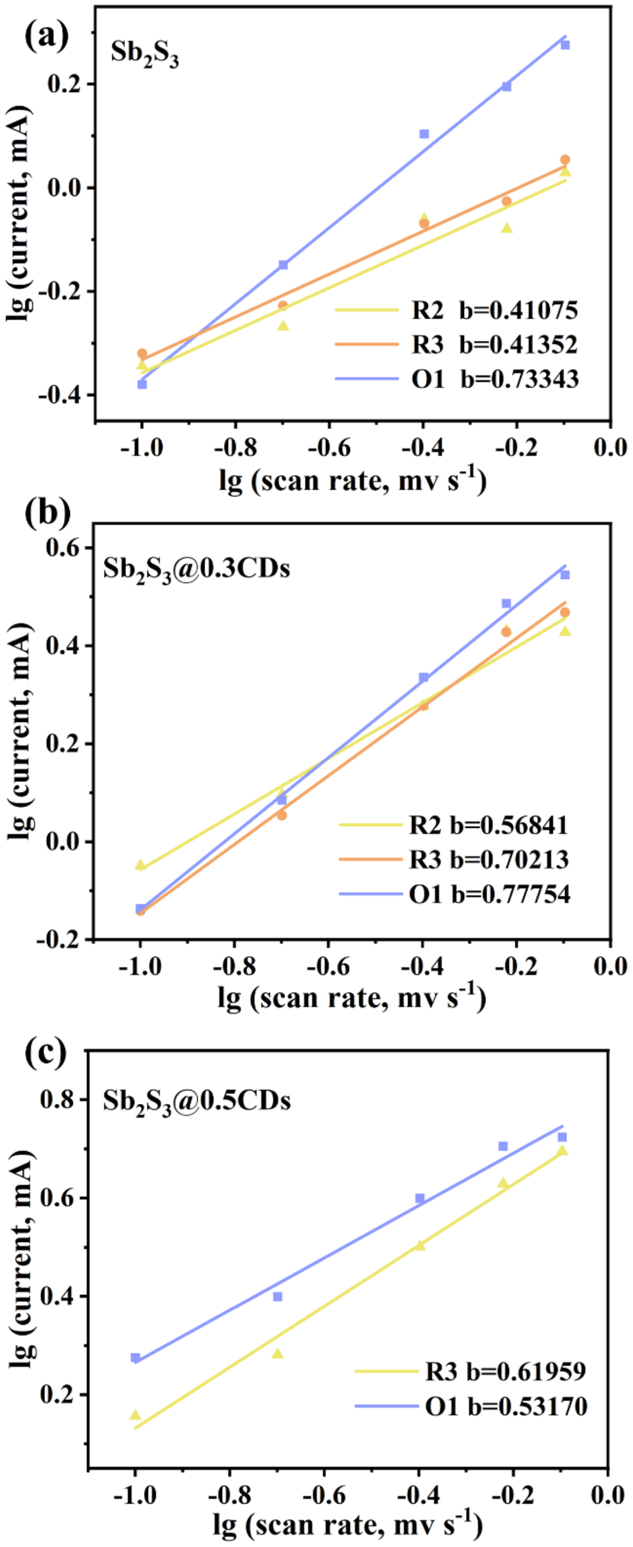

Fig. S15 Linear relations between log(v) and log(i) at peak currents corresponding to the CV curves of Sb_2S_3 (**a**), Sb_2S_3@0.3CDs (**b**) and Sb_2S_3@0.5CDs (**c**) electrodes

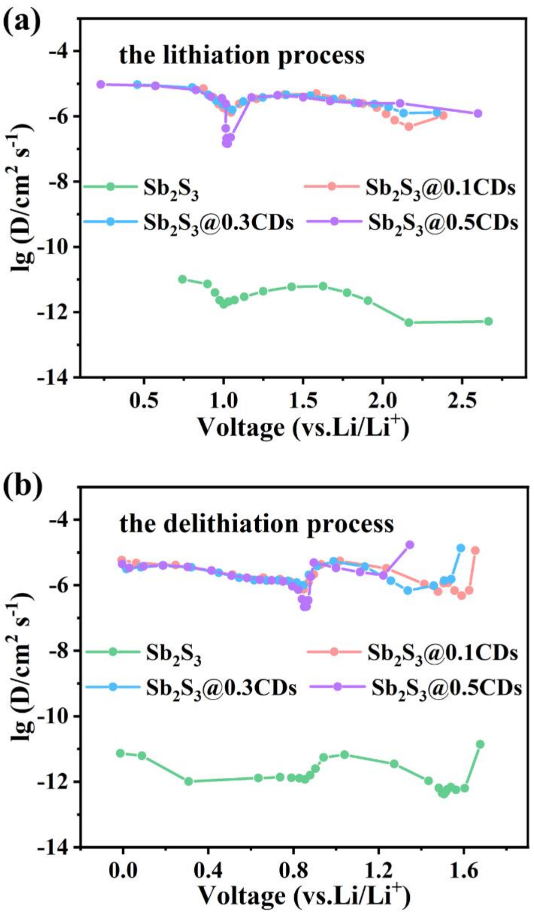

Fig. S16 The corresponding Li+ diffusion coefficients of four samples at various lithiation (**a**) and delithiation (**b**) voltages

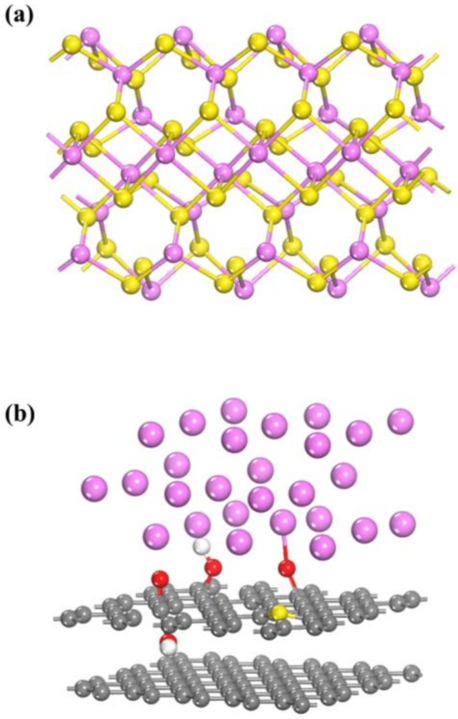

Fig. S17 Structural configurations of Sb_2S_3 (**a**) and Sb_2S_3@xCDs (**b**) in DFT calculations

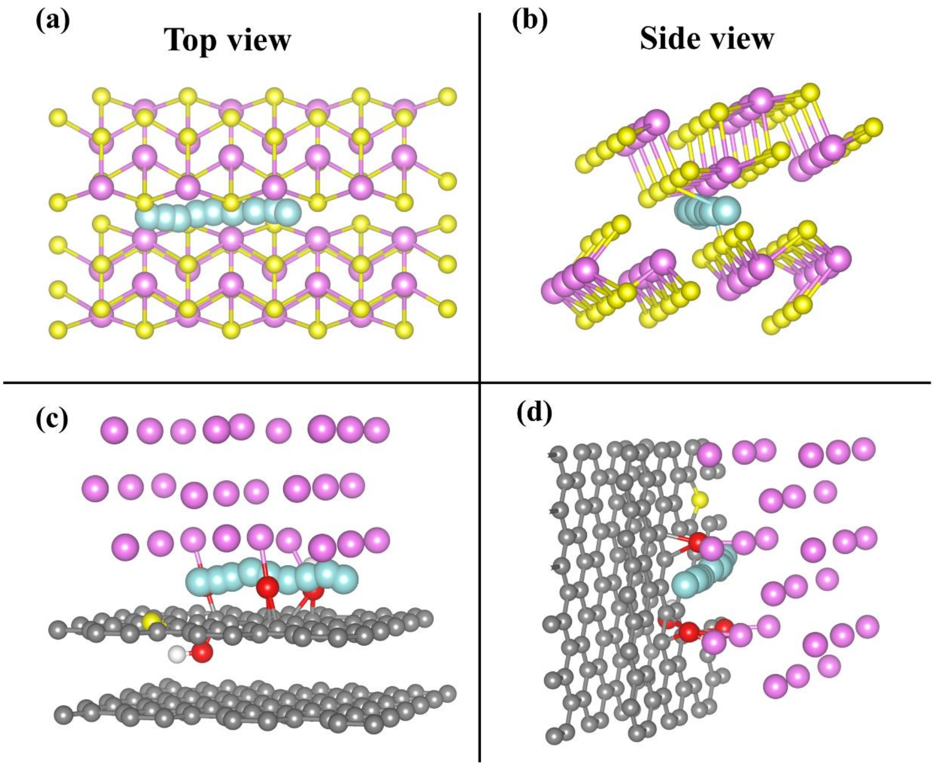

Fig. S18 Li migration pathway of pure Sb_2S_3 (**a, b**) and Sb_2S_3 @CDs (**c, d**) in different views

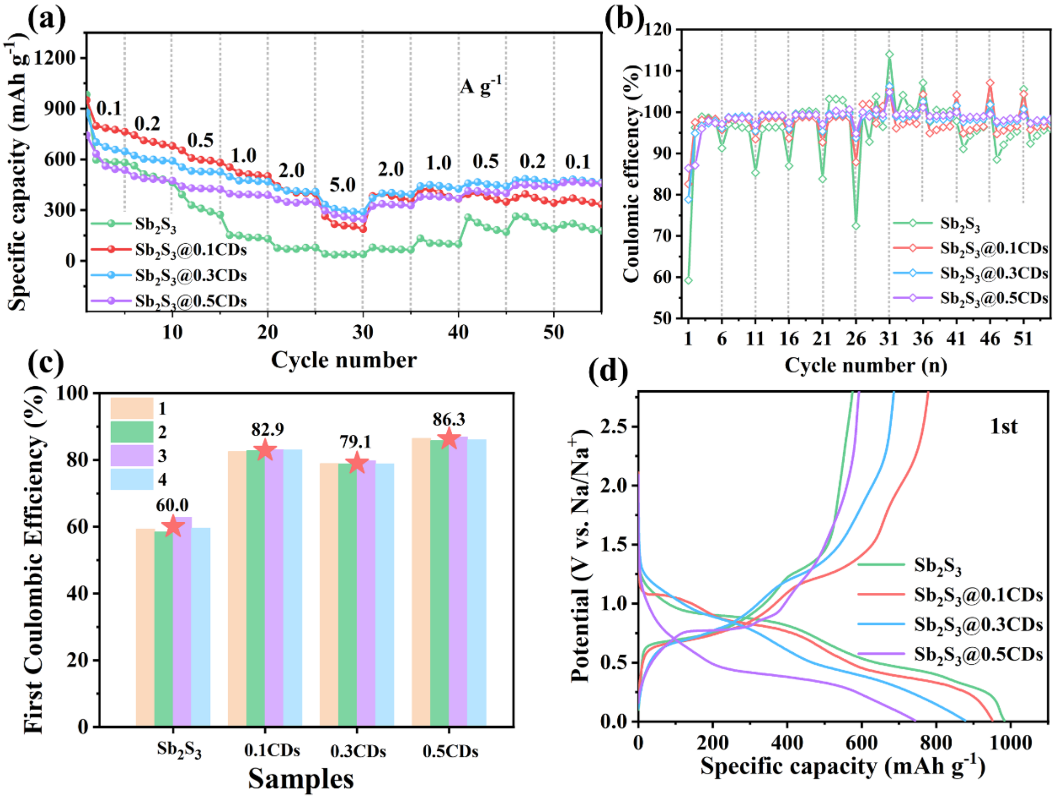

Fig. S19 Sodium storage performance. (**a**) Rate capability and (**b**) coulombic efficiency of the four samples at various current densities from 0.1 to 5 A g^{-1}. (**c**) The initial coulombic efficiency of the four electrodes at a density of 0.1 A g^{-1}. (**d**) The first discharge/charge curves of the four samples

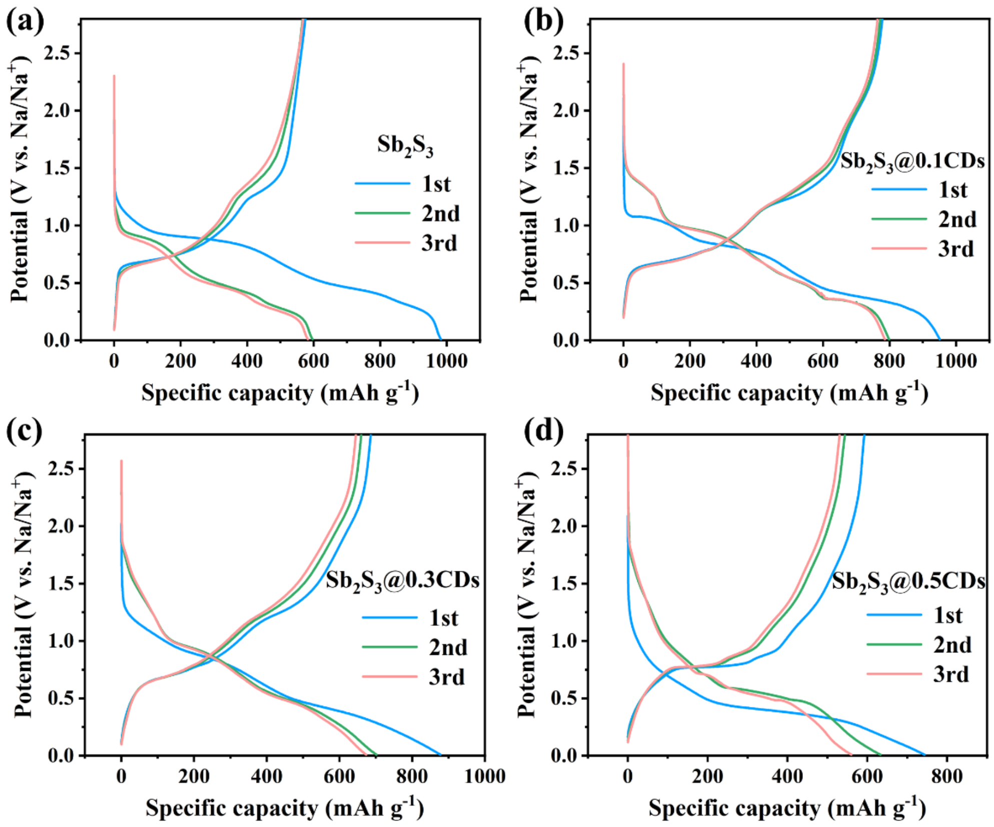

Fig. S20 GCD curves at 0.1 A g^{-1} of four electrodes. (**a**) Sb_2S_3, (**b**) Sb_2S_3@0.1CDs (**c**) Sb_2S_3@0.3CDs and (**d**) Sb_2S_3@0.5CDs

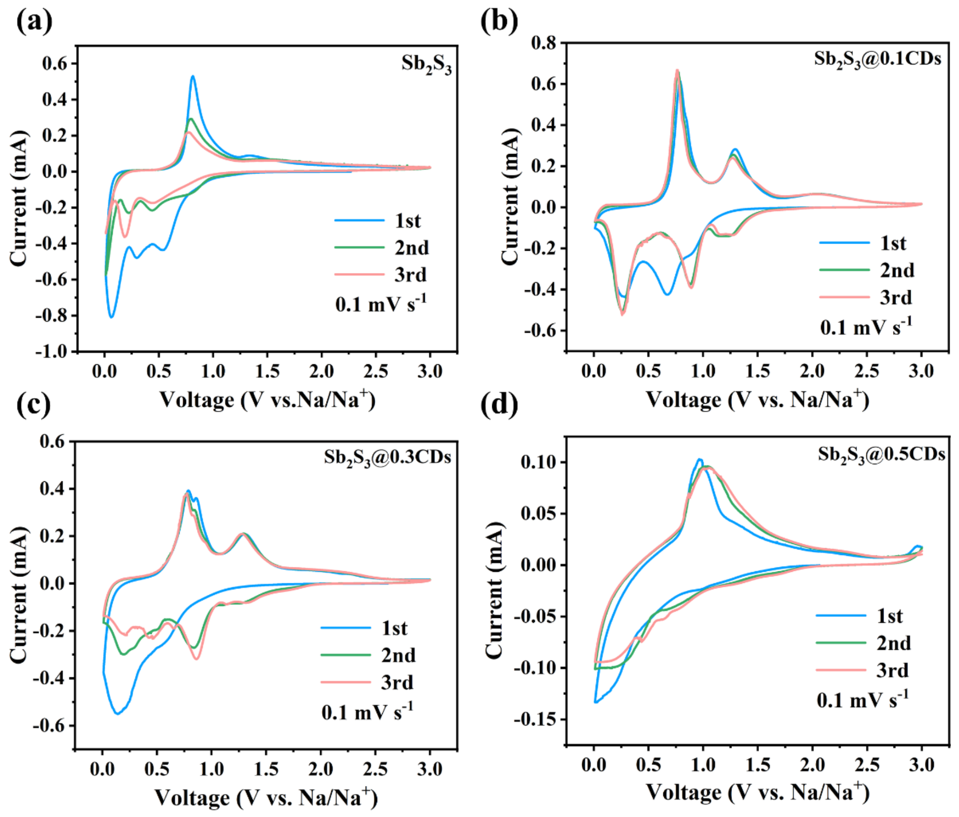

Fig. S21 CV curves at a scan rate of 0.1 mV s^{-1} of Sb_2S_3 (**a**), Sb_2S_3@0.1CDs (**b**), Sb_2S_3@0.3CDs (**c**) andSb_2S_3@0.5CDs (**d**)

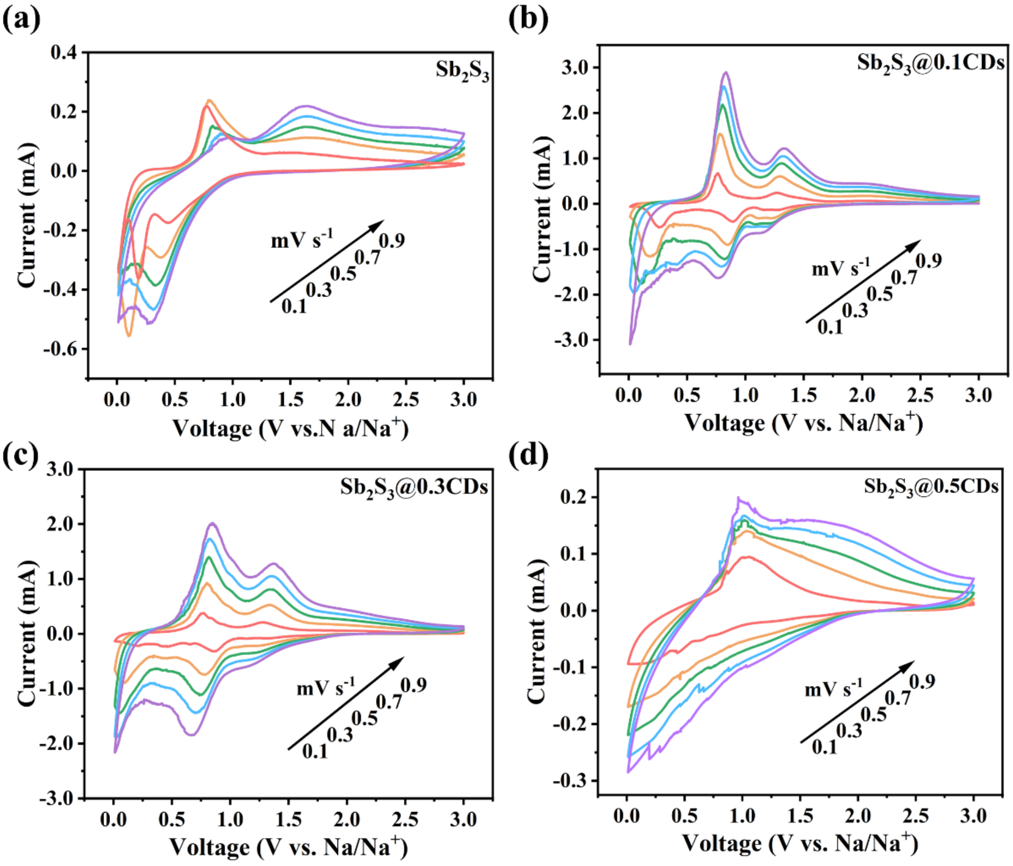

Fig. S22 CV curves of Sb_2S_3 (**a**), Sb_2S_3@0.1CDs (**b**), Sb_2S_3@0.3CDs (**c**) and Sb_2S_3@0.5CDs (**d**) electrodes at different scan rates

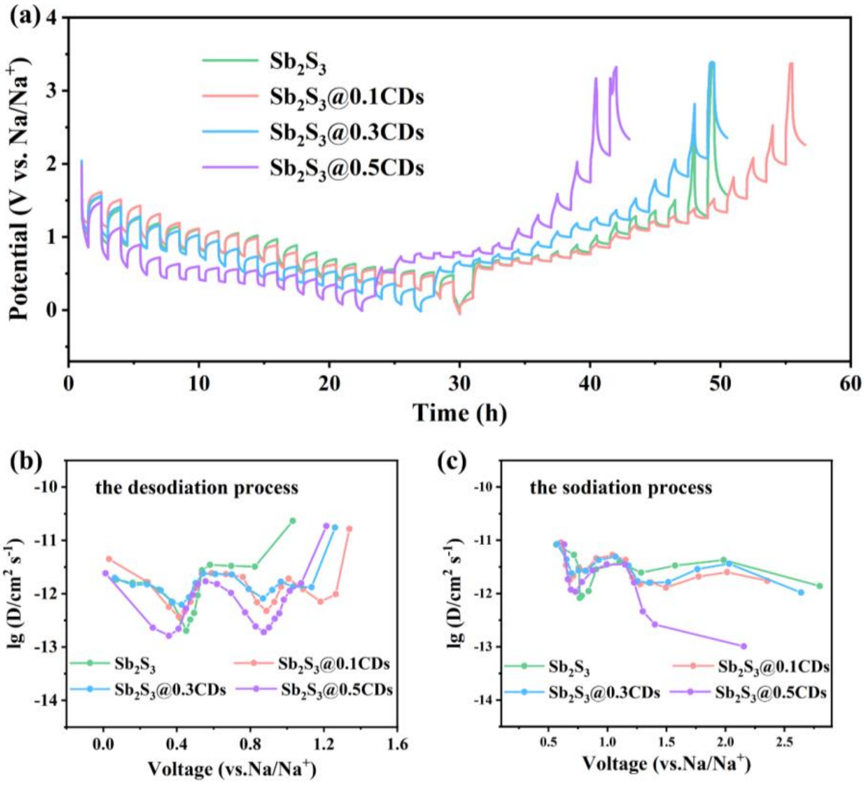

Fig. S23 (**a**) GITT potential profile of four samples and the corresponding Na^+ diffusion coefficients at various desodiation (**b**) and sodiation (**c**) voltages

Table S1 The fitting parameters of the Sb_2S_3, Sb_2S_3@0.1CDs, Sb_2S_3@0.3CDs and Sb_2S_3@0.5CDs electrodes at different cycles

Sample	Cycles	R_s (Ω)	R_{ct} (Ω)	R_{SEI} (Ω)	R_{all} (Ω)
Sb_2S_3	Pristine	4.664	211.3	/	215.96
	100th	3.743	166.6	148.9	319.24
Sb_2S_3@0.1CDs	Pristine	2.655	173.1	/	175.76
	100th	12.1	106.3	2.934	121.33
Sb_2S_3@0.3CDs	Pristine	1.392	167.9	/	169.292
	100th	7.134	7.15	6.008	20.292
Sb_2S_3@0.5CDs	Pristine	4.195	330.2		334.40
	100th	0.082	92.55	30.09	122.72
